# Rubber Degrading Strains: *Microtetraspora* and *Dactylosporangium*

**DOI:** 10.3390/polym13203524

**Published:** 2021-10-13

**Authors:** Ann Anni Basik, Jayaram Nanthini, Tiong Chia Yeo, Kumar Sudesh

**Affiliations:** 1 Ecobiomaterial Research Laboratory, School of Biological Sciences, Universiti Sains Malaysia, George Town 11800, Malaysia; annbasik@sbc.org.my; 2 Sarawak Biodiversity Centre, Km. 20 Jalan Borneo Heights, Kuching 93250, Malaysia; cyeo@sbc.org.my; 3 Faculty of Arts & Science, School of Science & Psychology, International University of Malaya-Wales, Kuala Lumpur 50480, Malaysia; nanthini.jayaram@iumw.edu.my

**Keywords:** biodegradation, *Dactylosporangium*, genome, latex clearing protein, *Microtetraspora*

## Abstract

Rubber composed of highly unsaturated hydrocarbons, modified through addition of chemicals and vulcanization are widely used to date. However, the usage of rubber, faces many obstacles. These elastomeric materials are difficult to be re-used and recovered, leading to high post-consumer waste and vast environmental problems. Tyres, the major rubber waste source can take up to 80 years to naturally degrade. Experiments show that the latex clearing proteins (Lcp) found in Actinobacteria were reportedly critical for the initial oxidative cleavage of poly(*cis*-1,4-isoprene), the major polymeric unit of rubber. Although, more than 100 rubber degrading strains have been reported, only 8 Lcp proteins isolated from *Nocardia* (3), *Gordonia* (2), *Streptomyces* (1), *Rhodococcus* (1), and *Solimonas* (1) have been purified and biochemically characterized. Previous studies on rubber degrading strains and Lcp enzymes, implied that they are distinct. Following this, we aim to discover additional rubber degrading strains by randomly screening 940 Actinobacterial strains isolated from various locations in Sarawak on natural rubber (NR) latex agar. A total of 18 strains from 5 genera produced clearing zones on NR latex agar, and genes encoding Lcp were identified. We report here lcp genes from *Microtetraspora* sp. AC03309 (*lcp1* and *lcp2*) and *Dactylosporangium* sp. AC04546 (*lcp1*, *lcp2*, *lcp3*), together with the predicted genes related to rubber degradation. In silico analysis suggested that *Microtetraspora* sp. AC03309 is a distinct species closely related to *Microtetraspora glauca* while *Dactylosporangium* sp. AC04546 is a species closely related to *Dactylosporangium sucinum*. Genome-based characterization allowed the establishment of the strains taxonomic position and provided insights into their metabolic potential especially in biodegradation of rubber. Morphological changes and the spectrophotometric detection of aldehyde and keto groups indicated the degradation of the original material in rubber samples incubated with the strains. This confirms the strains’ ability to utilize different rubber materials (fresh latex, NR product and vulcanized rubber) as the sole carbon source. Both strains exhibited different levels of biodegradation ability. Findings on tyre utilization capability by *Dactylosporangium* sp. AC04546 is of interest. The final aim is to find sustainable rubber treatment methods to treat rubber wastes.

## 1. Introduction

There is a great interest in developing environmentally sustainable methods to solve the rubber waste problem globally. Rubber consisting of poly(*cis*-1,4-isoprene) or poly(*trans*-1,4-isoprene) are highly modified through compounding and vulcanization in industrial processes and are used in more than 50,000 products today [1,2]. Rubber materials are resistant to thermal and chemical degradation. They are also difficult to re-use or recycle. And the majority of them are just discarded in landfills [3,4,5]. Robust, economical, and easily scalable methods for converting rubber products or waste into by-products or composites that ideally can be reused are desperately needed.

Rubber-degrading enzymes, produced by microbes, are a key discovery for biological rubber degradation. They consist of latex clearing protein (Lcp), mainly present in Actinobacteria and rubber oxygenases (RoxA and RoxB) found in Gram negative rubber degraders. There are 2 groups of rubber-degrading bacteria: (i) strains that produce clear zones on NR latex agar and (ii) strains that require direct contact with rubber substrates for subsequent degradation [6,7,8]. In both cases, enzymes produced by the bacteria extracellularly cleave rubber polymers containing hydrocarbon with a molecular weight (Mw) of 10^5^ to 10^6^ into mixtures of low Mw products C_20_, C_25_, C_30_ and higher oligo-isoprenoids as end products [9,10,11]. Oligomers with an Mw higher than 2000 were slowly degraded by microbes, while short oligomers of roughly 1000 Mw were rapidly consumed by the organism [12].

Actinobacteria are an integral part of the indigenous soil microflora, unique for their ability to degrade a large variety of organic materials, their high catabolic capacity, and their resilience in the face of unfavorable environmental conditions [13,14]. The same species of Actinobacteria growing in different locations or ecological niches are known to produce environmentally specific metabolites [15,16]. Biodiversity in Sarawak is a great advantage in our field of study. Actinobacteria that have evolved genes encoding catabolic enzymes that are functionally capable of degrading modified rubber are a potential solution for the end-of-life management of rubber products [4,17,18].

Our present survey of the Sarawak Biodiversity Centre (SBC) (https://www.sbc.org.my/, accessed on 5 June 2021) Actinobacteria Culture Collection is geared towards discovering novel rubber-degrading Actinobacteria. Most rubber-degrading strains discovered to date belong to the phylum, Actinobacteria (mainly *Streptomyces*, *Gordonia*, *Nocardia* and *Rhodococcus*) and are rarely found among Gram-negative bacteria and fungi [4,19,20,21]. Furthermore, several findings proved that different rubber-degrading strains possess different rubber-degrading ability. *Nocardia* genus, in particular *Nocardia nova* SH22a, has been characterized for its ability to degrade both poly(*cis*-1,4-isoprene) and poly(*trans*-1,4-isoprene) rubber [22,23]. The biochemical study of Lcp from *Rhodococcus rhodocrous* RPK1 showed substantial differences in its active site compared to Lcp of *Streptomyces* sp. K30 and *Gordonia polyisoprenivorans* VH2 [24]. In addition to that, a combination of different rubber oxygenases (RoxA/RoxB/Lcp) had a synergistic effect on rubber degradation [9]. The degradation rate of rubber products was also greatly improved when utilizing a consortium of rubber-degrading strains instead of using a specific strain [25,26]. To augment existing information and evaluate the diverse activities in these groups of bacteria, findings, and the characterization of new rubber degraders and their metabolic capacity, are required.

To date, a total of 248 lcp genes have been deposited into GenBank. This screening program led to the discovery of 2 new rubber-degrading genera, *Microtetraspora* and *Dactylosporangium* containing rubber-degrading genes. Limited information is available for both genera due their low isolation frequency. Classified as “rare Actinobacteria” only 22 and 16 Type strains have been published to date for *Microtetraspora* and *Dactylosporangium*, respectively [27]. There have been no reports on the biodegradation ability of *Microtetraspora* and *Dactylosporangium,* and their genome-based characterization as rubber degraders.

## 2. Materials and Methods

This research was carried out by firstly detecting rubber-degrading strains by screening them on natural rubber (NR) latex agar. Clear zone formation on NR latex agar indicated the ability of the strain to utilize NR latex. These strains were then morphologically and molecularly characterized. Based on the strain taxonomic and latex-clearing protein (lcp) genes characterization information, strains of interests of *Microtetraspora* sp. AC03309 and *Dactylosporangium* sp. AC04546 were selected for further studies. In silico studies of their genome through genomic sequencing were used to determine the strains’ novelty (taxonomic position, rubber-degrading genes and specialty genes). Both strains were also tested for their ability to utilize NR and vulcanized rubber (VR) as the sole carbon and energy source. The research approach flow chart is depicted in Supplementary Figure S1.

### 2.1. Screening for Clear Zone Forming Strains on Natural Rubber (NR) Latex Agar

Natural rubber (NR) latex agar preparation: the NR Latex agar preparation was modified from Braaz et al., 2004. [28] Mineral Salts Medium (MSM) per litre [K_2_HPO_4_, 8.0 g; KH_2_PO_4_, 1.0 g; (NH_4_)_2_SO_4_, 0.5 g; MgSO_4_·7H_2_O, 0.2 g; NaCl, 0.1 g; Ca(NO_3_)_2_, 0.1 g; 15 g agar] were added with 0.02% (*v*/*v*) NR latex concentrate and was sterilized by autoclaving for 15 min at 121 °C, 15 psi. After sterilization, 1 mL of 100× trace element solution was added.

NR latex concentrate: Crude rubber latex consists of 6% non-rubber constituents, mainly protein. High speed centrifugation was used to remove protein from freshly collected rubber latex. Fresh latex was transferred into high-speed polycarbonate centrifuge tube and equal volume of 0.002 % (*v*/*v*) Tween 80 was added, mixed well and then separated by centrifugation (5 min at 19,320× *g*). This washing step was repeated. The top layer or NR latex concentrate was removed and used to prepare the NR latex agar [29].

Trace elements (100×) per 100 mL [containing CaCl_2_·2H_2_O, 0.2 g; FeSO_4_·7H_2_O, 0.2 g; Na_2_MoO_4_·H_2_O, 5 mg; and MnSO_4_, 5 mg] were filter-sterilized [30].

MSM agar with NR latex concentrate were then transferred into 6-well plates (4 mL per well). Actinobacterial cultures cultivated in Yeast Malt Extract (ISP2) broth [31] for 3 to 14 days were spotted (40 µL) onto each agar well. The plates were incubated at 28 °C for up to 4 weeks. Colonies that produced translucent clearing zones, indicating the degradation of the NR latex, were recorded.

### 2.2. Identification of Clear Zone Forming Strains on Natural Rubber (NR) Latex Agar

Strains producing clear zones were taxonomically identified based on their morphological features and molecular techniques. Strains that were cultivated in ISP2 agar plate were used to capture and describe their morphology. Strains that were cultivated on Soil Extract agar (SEA) were used to observed the strains spore-forming structures [32]. SEA agar promotes the strains formation of spores and are translucent, easy for direct observation using a 50× long distance (Olympus LMPLFLN, Tokyo, Japan). Molecular identification was made based on the amplification of the 16S rDNA gene using primer 27F (5′-AGAGTTTGATCMTGGCTCAG-3′) and 1492R (5′-TACGGYTACCTTGTTACGACTT-3′) with the following parameters: 5 min at 96 °C, 30 cycles of 45 s at 96 °C, 2 min at 55 °C, 4 min at 72 °C and the final extension for 7 min at 72 °C. The amplified products were sent to Apical Scientific Sdn. Bhd., Selangor, Malaysia for sequencing. Sequence quality was checked using Sequence scanner version 2.0 (Applied Biosystems, Waltham, MA, United States) [33] and assembled by manual alignment (cap contig) using BioEdit software version 7.0.5.3 (North Carolina State University, Raleigh, NC, USA) [34]. The sequences were subjected to online BLASTN analysis against the 16S ribosomal RNA sequences (Bacteria and Archaea). Phylogenetic tree was generated using MEGA X software (Penn State University, State College, PA, USA) [35].

### 2.3. Profiling of Latex Clearing Protein (Lcp) Genes

The presence of lcp genes in the strains that produced clearing zones on NR agar were screened and amplified using the following primers: lcp441f [5′-GGAG(TG)C(GC)GC(GC)GTCTACTACTC-3′] and lcp879r [5′-GATCGG(AG)T(TC)GAG(AGC)ACCTGC-3′] [30], lcp1f [5′-ATGGAGAATCTCAGTAGACGT-3′] and lcp1199r [5′-ATGACCGGAATGGTGATCGG-3′], designed based on the conserved regions of published lcp gene sequences (*Micromonospora* sp. WMMB235 (ENA accession number MDRX01000001), *Micromonospora* sp. TSRI0369 (ENA accession number LIVU01000002), *Micromonospora* sp. NRRL B-16802 (ENA accession number NZLGEB01000064), *Micromonospora echinospora* ATCC15837 (ENA accession number NGNT01000005). The target genes were amplified using the following parameters: 2 min at 95 °C, 30 cycles of 30 s at 95 °C, 1 min at 55–65 °C, 2 min at 72 °C and the final extension for 5 min at 72 °C. Annealing temperature was optimized for each strain.

Amplified products were sent to Apical Scientific Sdn. Bhd., Selangor, Malaysia for sequencing. Sequence quality was checked using a Sequence scanner version 2.0 (Applied Biosystems, Waltham, MA, USA) [33] and assembled by manual alignment (cap contig) using BioEdit software version 7.0.5.3 (North Carolina State University, Raleigh, NC, USA) [34]. The sequence’s blast homology was compared to the UniProt database [36]. Nucleotide sequences were translated into amino acids using ExPASy (SIB Swiss Institute of Bioinformatics, Lausanne, Switzerland) [37] to identify the open reading frame (ORF). ORF were subjected to BlastP (GenBank, MD, USA) to obtain Protein Blast homology.

### 2.4. Genomic Deoxyribonucleic Acid (DNA) Isolation

Genomic DNA (gDNA) for *Microtetraspora* sp. AC03309 and *Dactylosporangium* sp. AC04546 were extracted and purified using an adapted method from Moore et al., (2008) [38]. Strains were first cultivated in 20 mL ISP2 broth in a 250 mL flask at 28 °C, 180 rpm for 7 to 10 days. Harvested Actinobacterial cells were centrifuged and washed with sterile water twice to remove any growth media. The cells (~0.1 g) in PCR tubes were then resuspended in 550 µL 1xTris-EDTA buffer and mixed well. Lysozyme (for cell lysis) with final concentration of 2 mg mL^−1^ and 5 µL Rnase A solution (10 mg mL^−1^) were added and incubated at 37 °C for 90 min. Then 10% (*v*/*v*) SDS (final concentration 1% *v*/*v*) and Proteinase K 10 mg mL^−1^ (final concentration 1 mg mL^−1^) were added and incubated at 65 °C for 90 min. SDS is an anionic detergent which binds cellular proteins and lipoproteins, effectively denaturing them and promoting the dissociation of nucleic acids. 5M NaCl was added (final concentration 0.7 M) and incubated at 65 °C for 2 min, then pre-warmed CTAB (hexadecyltrimethylammonium bromide) (final concentration 1% *v*/*v*) was added and incubated at 65 °C for another 10 min. CTAB has also been proven useful for DNA extractions from bacterial cells by denaturing and precipitating the cell wall lipopolysaccharides and proteins. In the presence of monovalent cation (e.g., Na+) concentrations above 0.5 M, DNA will remain soluble.

To purify the gDNA sample, equal volume (*v*/*v*) of CIA (24: 1, chloroform: isoamyl alcohol) was added, inverted to mix, and centrifuge at 10,000 rpm for 5 min. This step was repeated twice. The top layer was transferred and added with equal volume of isopropanol. The sample is kept at −20 °C overnight to allow gDNA to precipitate. Genomic DNA was pellet by centrifuging at 14,000 rpm for 15 min. Isopropanol was removed, and the gDNA pellet washed with 70% molecular grade ethanol. Genomic DNA was pellet by centrifuging at 14,000 rpm for 15 min. Ethanol was discarded and the tube containing gDNA pellet was left to air dry. Finally, the gDNA was resuspended in 10 mM Tris-HCL buffer pH8.5. DNA quantity and quality was checked using BioSpectrophotometer (Eppendorf AG, Germany).

Genomic DNA was then analyzed for its integrity and purity using gel electrophoresis. A minimum amount of 200 ng gDNA eluted in 30 µL Tris-HCl buffer (DNA concentration 6 µg) was required for library preparation (Nextera XT Library Prep Kit, Illumina, CA, USA) prior to sequencing. The purified DNA samples were subsequently sent for genomic sequencing using Illumina MiSeq sequencer (Illumina, CA, USA) and were assembled (SPAdes) by service provider BioEasy Sdn. Bhd., Selangor, Malaysia.

Genomic DNA extraction was also carried out for all the clear zone forming strains for 16S rRNA and latex-clearing protein (lcp) gene amplification using polymerase chain reaction (PCR).

### 2.5. Genome Attributes and Annotation

Draft genome for *Microtetraspora* sp. AC03309 and *Dactylosporangium* sp. AC04546 were annotated using RAST server version 2.0 [39], PATRIC server version 3.6.12 [40] and NCBI Prokaryotic Genome Annotation Pipeline (PGAP) [41]. Gene function annotation for Clusters of Orthologous Groups (COGs) were carried out using egg-NOG mapper v.2 [42]. COG retain the same function and are orthologous across at least three lineages, likely corresponds to an ancient, conserved domain [43]. To determine the related genes/protein and location in a pathway, the KEGG Pathway database was used [44]. Clustered regularly interspaced short palindromic repeats (CRISPRs) were used to depict the genome readability for bacteriophage exposure and genome-editing [45]. To predict the antibiotic-resistant genes, antibiotic-resistant homologs search was carried out in the CARD database (https://card.mcmaster.ca/analyze/rgi, accessed on 5 June 2021) [46]. Drug targets were identified using PARTIC server [40] and verified using DrugBank 5.0 [47].

The strains’ ability to produce secondary metabolites was predicted using The Antibiotics and Secondary Metabolite Analysis Shell (AntiSMASH) 6.0 software [48].

To determine the location of protein-coding genes (CDS) and open reading frame (ORF) on the draft genome, sequence contigs were viewed using The SEED Viewer [49] and SnapGene Viewer (https://www.snapgene.com/, accessed on 5 March 2020).

CDS and amino acid sequence for latex clearing protein (Lcp), 1-oxidoreductase beta subunit (oxiB) and 1-oxidoreductase alpha subunit (oxiA) were verified through the presence of ribosome-binding site (RBS), and comparison with sequences in UniProt [50] and GenBank database. The prediction of twin arginine translocation (Tat) signal peptides was carried out using TatP 1.0 server (Department of Health and Technology, Lyngby, Denmark) [51]. ExPASy was used to determine the predicted protein molecular weight (Mw) and their theoretical isoelectric point (pI) [37].

### 2.6. Taxonomic Delineation

The taxonomic position of *Microtetraspora* sp. AC03309 and *Dactylosporangium* sp. AC04546 was determined using polyphasic approaches, including (i) 16S rRNA gene-based method, (ii) average nucleotide identity (ANI) using FastANI (KBase Software, U.S. Department of Energy, Washington, DC, USA) [52], (iii) in silico DNA-DNA hybridization (dDDH) estimate method and (iv) genome relatedness and phylogenetic analysis. Both dDDH and phylogenetic analysis (16S rRNA and genome) was conducted using Type (Strain) Genome Server (TYGS) (DSMZ, Braunschweig, Germany) [53]. A minimum of 20% of the genome is required to obtain the same result as the full genome [54]. Better-resolved phylogenies, based on the hundreds of housekeeping genes or even the core-genome, has been used to elucidate the phylogenetic relationships classifications based on whole genome sequences [55].

### 2.7. Utilization of Rubber Materials

To study the ability of *Microtetraspora* sp. AC03309 and *Dactylosporangium* sp. AC04546 in utilizing different rubber materials as the sole carbon and energy source; fresh latex, rubber gloves and tyres samples were used. Fresh latex was harvested from 5-year-old rubber trees at a rubber plantation site at Kulim, Kedah, Malaysia. The latex was brought back and left to solidify at room temperature. Solid latex pieces were then cut into ~1.0 cm × 1.0 cm pieces. Steel-free tyre granules (1.0 to 3.0 mm) were obtained from a tyre recycling factory (Gcycle Tyre Recycling) in Bedong, Kedah, Malaysia. Latex gloves (PRO-CARE), disposable and non-powdered, were used in these studies. Latex gloves were cut into strips of ~0.5 cm × 1.0 cm.

For short-term evaluation under laboratory conditions, antimicrobial substances from the latex glove and tyre granules were removed prior to cultivation. The rubber materials were treated with chloroform as follows: 1 g of sample with 100 mL for 12 h. During this period the solvent was replaced one to two times with fresh chloroform. The treated material was left to air dry; then, it was sterilized by autoclaving and subsequently used as carbon source [56]. No changes in the surface (cracks and holes) of the rubber material were observed using Scanning Electron Microscope (SEM) (Quanta FEG 650, Thermo Fisher Scientific, Edinburgh, UK) before and after chloroform treatment.

Pre-culture of actively growing strains were cultivated in ISP2 broth. The culture (1 mL) were transferred into 250 mL test flasks containing 50 mL MSM and 0.5% (*w*/*v*) rubber material [56]. The inoculated flasks were then incubated at 28 °C, 180 rpm for 30 days. Test flasks without culture were used as control. All the sample studied were carried out in triplicates.

- Morphological Observation using Scanning Electron Microscope (SEM)

Inoculated rubber samples were harvested and air-dried for direct observation. To observe the surface of the tested rubber material, the strain biofilm and mycelia on the rubber particles were removed by rinsing the rubber materials with distilled water. Then, immersing them in 96% ethanol (Sigma-Aldrich, Switzerland) for 1 h before air-drying at ambient temperature. Both washed and unwashed samples were prepared using the hexamethyldisilazane (HDMS, Sigma-Aldrich, Switzerland) method prior to SEM viewing. Samples were placed into shell vials (1.8 mL) and fixed by immersing it in *McDowell-Trump* fixative (Thermo Fisher Scientific, Waltham, MA, USA) solution, prepared in 0.1 M phosphate buffer (pH 7.2) (Sigma-Aldrich, Switzerland) at 4 °C for at least 2 h. The samples were then washed in 0.1 M phosphate buffer (pH 7.2) (Sigma-Aldrich, Switzerland) for 10 min, this step is repeated twice. Samples were post-fixed by immersing them in 1% (*v*/*v*) osmium tetroxide prepared in 0.1 M phosphate buffer (Sigma-Aldrich, Switzerland) (pH 7.2) for 1 h. This step was carried out in the chemical hood. Then the samples were washed by immersing them in distilled water for 10 min. This step was repeated twice. Dehydration of the samples was carried out using ethanol. Firstly, the samples were immersed in 50% ethanol (Sigma-Aldrich, Switzerland) for 15 min, followed by 75% for 15 min, 95% for 15 min (twice) and finally 100% for 20 min (3 times). The dehydrated samples were then immersed in 1 mL HDMS for 10 min. HDMS acts as a drying agent. HDMS solutions were discarded, and the samples were left to dry in the desiccator. The dried samples were then mounted onto an SEM stub using double tape, coated with gold using Quorum Q150T S sputter coater (Quorum Technologies Ltd., East Sussex, UK) for observation using SEM Quanta FEG 650 (Thermo Fisher Scientific, Edinburgh, UK).

- Attenuated Total Reflection-Fourier Transform Infrared (ATR-FTIR)

Strain biofilm and mycelia were removed from the tested rubber samples by rinsing them with distilled water. Samples were then immersed in 96% ethanol for 1 h before air-drying them at room temperature. FTIR-ATR was used to determine the formation of new, or disappearance of functional groups in the polymer units of the samples by observing the presence, increase and decrease in C=C, C-C and C-H bonds. Post-incubation samples (fresh latex, latex glove, tyre) and non-inoculated samples were analyzed using ATR-FTIR Spectrum 400 (Perkim Elmer), equipped with ATR ranging from 4000 cm^−1^ to 650 cm^−1^, with 4 cm^−1^ resolution.

## 3. Results

### 3.1. Screening for Clear Zone Forming Strains on Natural Rubber (NR) Latex Agar

Since 2006, Sarawak Biodiversity Centre (SBC) has been collecting environmental samples from various locations across Sarawak in efforts to isolate microbes mainly Actinobacteria and fungi as part of their inventory. To evaluate the potential application of the Actinobacterial Culture Collection as rubber degraders, a total of 940 strains were randomly selected and screened on NR latex agar. The strains tested consisted of Actinobacteria from 9 families (15 genera) and 205 Actinobacteria strains that could not be classified based on morphology (Table 1). Eighteen (18) strains were found to be capable of degrading NR latex, producing ~1 to 2 mm clearance surrounding the colony.

### 3.2. Taxonomic Identification of Clear Zone Forming Strains

All of the 18 strains producing clear zones were successfully identified through PCR amplification of their 16S rDNA gene having blast homology ranging from 99.00 to 100.00% (MT005091, MT005089, MT005088, MT005098, MT005101, MT005095, MT005096, MT005104, MT005094, MT005105, MT005103, MT005100, MT005099, MT005102, MT005097, MT005093, MT005090, MT005092). Blast homology of 98.70–99.50% are putative novel species, 99.60–99.80% are putative known species and 99.90% and above are identical or closely related species [57].

Clear zone forming strains were distributed among 5 genera: *Microtetraspora* sp. (7 strains), *Micromonospora* sp. (6 strains), *Streptomyces* sp. (2 strains), *Nonomuraea* sp. (2 strains) and *Dactylosporangium* sp. (1 strain). Although 39% of clear zone formers identified in this study belong to *Microtetraspora* genus, phylogenetic distribution of 16S rRNA gene suggests that they are not identical (Supplementary Figure S2).

### 3.3. Profiling of Latex Clearing Protein (Lcp) Genes in Clear Zone Forming Strains

Latex-clearing protein (Lcp) is responsible for the formation of clear zones on natural rubber (NR) overlay agar plates [58]. Blast homology of the amplified gene sequence was compared to the UniProt database, resulting in 81.20 to 100.00% blast identity to closely related proteins. The amplified nucleotide sequences were curated and submitted to GenBank with the following accession numbers: MN148090, MT241322, MN148093, MN148092, MN148094, MN148095, MT252675, MN148097 MN148098, MT241320, MN148096, MT241323, MT241319, MT241318, MT241317, MT252676, MT241321, MN148089.

Lcp amino acid sequences have been reported to contain 13-residues-long highly conserved region “KTRLVHAAVRHLL” [59]. ClustalW multiple alignment of the amplified Lcp amino acid sequences obtained in this study contained the highly conserved region, except for *Micromonospora* sp. AC03293 and *Streptomyces* sp. AC04842 (Supplementary Figure S3). Although the conserved region was not detected for strain *Micromonospora* sp. AC03293 due to its short sequence, the Lcp blast homology was 95.90% identity to *Micromonospora* sp. WMMB235 *lcp* gene. A comparison of Lcp amino acid sequence for *Micromonospora* sp. AC03293 and biochemically characterized Lcp from *Nocardia nova* SH22a also showed close similarity (85.70%). PCR amplification of *Streptomyces* sp. AC04842 *lcp* gene region was unsuccessful. Blast homology shows that it was 85.40% identical to a hypothetical protein of *Streptomyces* sp. 4F (QIS63111.1). However, in a separate study, we were able to identify *lcp*-homologues (MT664881 and MT664882) in the draft genome of *Streptomyces* sp. AC04842, containing the 13-residues-long highly conserved region.

### 3.4. Strain of Interest: Microtetraspora sp. AC03309 (JCM 34240)

The *lcp* gene and the genomic characterization for both genera, *Microtetraspora* and *Dactylosporangium* have not been reported. Therefore, we chose to further explore them through: (i) morphological studies and taxonomic delineation, (ii) prediction of genes related to rubber degradation through genome sequencing, and (ii) their ability to utilize different rubber products as the sole carbon and energy source.

*Microtetraspora* sp. AC03309 was isolated from a soil sample collected in a forest nearby Kampung Kiding, Padawan, Sarawak in 2006.

#### 3.4.1. Morphological Characterization of *Microtetraspora* sp. AC03309

**Figure 1:**
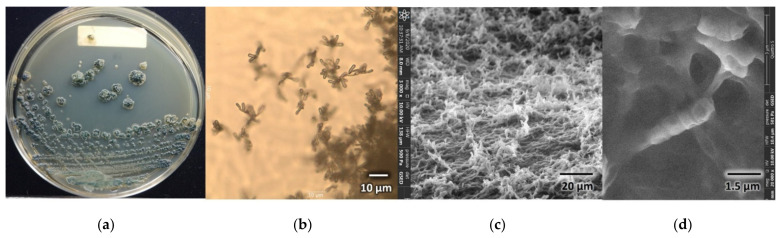
*Microtetraspora* sp. AC03309 morphology: (**a**) surface colony morphology on Yeast Malt Extract (ISP2) agar; (**b**) spore-forming structures viewed using light microscope at 750× magnification on Soil Extract agar (SEA); (**c**) sporophore branching from aerial mycelia viewed using Field Emission Scanning Electron Microscope (FESEM, Quattro Thermo Fisher Scientific, Lanham, MD, USA); (**d**) sporophore with four spores viewed using Field Emission Scanning Electron Microscope (FESEM, Quattro Thermo Fisher Scientific, Lanham, MD, USA).

Morphological characteristics of *Microtetraspora* sp. AC03309 can be seen in (Figure 1). *Microtetraspora* sp. AC03309 has blue green surface and honey gold reverse color on ISP2 agar with moderate aerial mycelia. Colony is wrinkled with regular shape; it has external short spore chains (approx. 3 µm length) with 4 spores formed on sporophore branching from aerial hyphae. Spores are slightly oval to cylindrical with smooth surfaces. It grows well at 28 °C, with poor growth at 40 °C and 45 °C on ISP2 agar.

#### 3.4.2. *Microtetraspora* sp. AC03309 Taxonomic Delineation

The complete 16S rRNA gene sequence for *Microtetraspora* sp. AC03309 (1477 bp) was used for online BLASTN analysis against the 16S ribosomal RNA sequences (Bacteria and Archaea). *Microtetraspora* sp. AC03309 had a 98.85% identity to *Microtetraspora glauca* strain IFO 14761 (NR_112346.1). The recommended threshold of >98.65% 16S rRNA gene sequence similarity is used as a threshold for differentiating two species [60].

**Figure 2:**
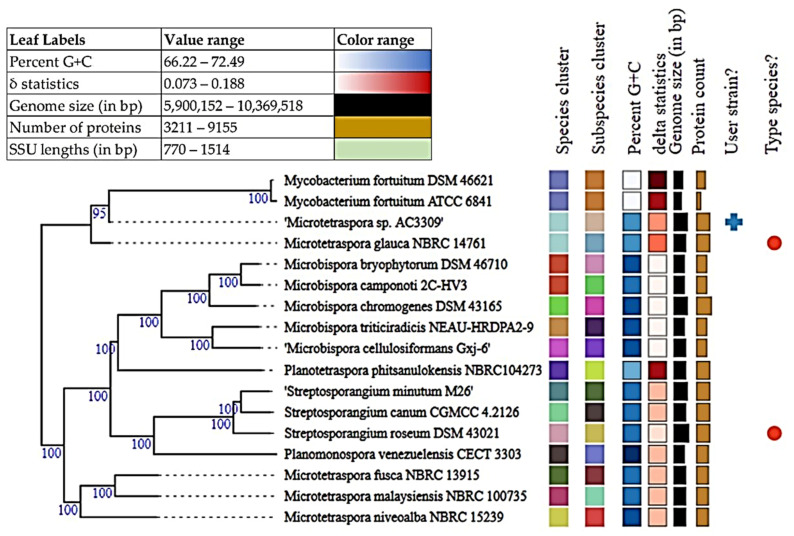
Tree inferred with FastME 2.1.6.1 (Lefort et al., 2015) from GBDP distances calculated from genome sequences. The branch lengths are scaled in terms of GBDP distance formula d5. The numbers above branches are GBDP pseudo-bootstrap support values > 60% from 100 replications, with an average branch support of 100%. The tree was rooted at the midpoint [62].

Currently, bacterial species are defined as including strains that have 95.00–96.00% of average nucleotide identity (ANI) and 70.00% of digital DDH (dDDH) [61]. Analysis between *Microtetraspora* sp. AC03309 and *Microtetraspora glauca* draft genome (TYGS, https://tygs.dsmz.de, accessed on 25 June 2021) provided an ANI estimate of 97.33%, digital DNA-DNA hybridization (dDDH) of 77.00%, together with genome-based phylogeny predicts that they belong to the same species (Figure 2). However, dDDH value of less than 79.00% suggests *Microtetraspora* sp. AC03309 strain to be of a distinct subspecies [53].

#### 3.4.3. *Microtetraspora* sp. AC03309 Genomic Attributes

- Genomic & Protein Features

**Figure A1:**
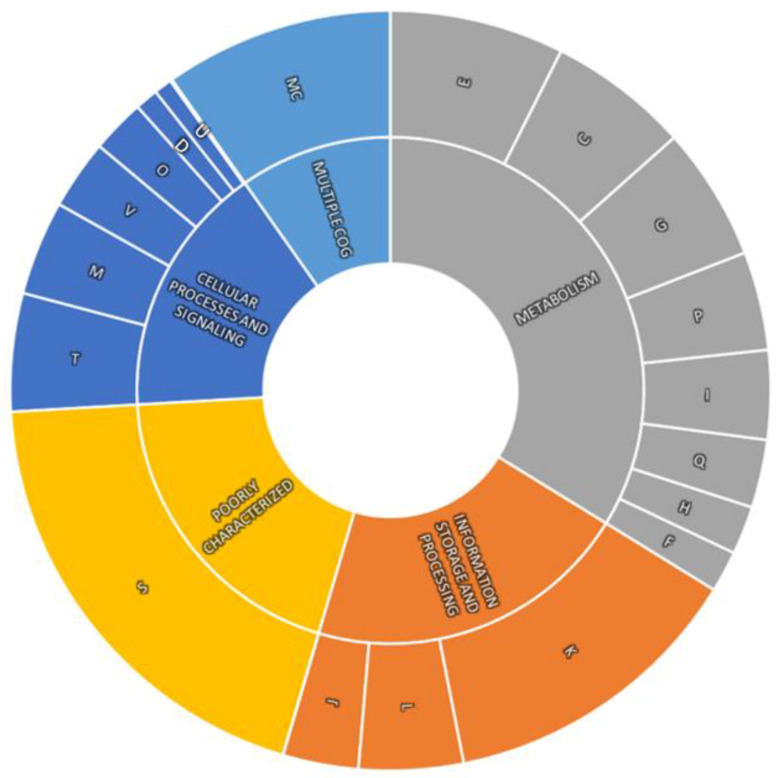
Cluster of Orthologous (COGs) for *Microtetraspora* sp. AC03309 analyzed using egg-NOG mapper v.2 [42]. CELLULAR PROCESSES AND SIGNALING [D] Cell cycle control, cell division, chromosome partitioning, [M] Cell wall/membrane/envelope biogenesis, [N] Cell motility; [O] Post-translational modification, protein turnover, and chaperones, [T] Signal transduction mechanisms, [U] Intracellular trafficking, secretion, and vesicular transport, [V] Defense mechanisms [W] Extracellular structures, [Y] Nuclear structure, [Z] Cytoskeleton; INFORMATION STORAGE AND PROCESSING [A] RNA processing and modification, [B] Chromatin structure and dynamics, [J] Translation, ribosomal structure and biogenesis, [K] Transcription, [L] Replication, recombination and repair; METABOLISM [C] Energy production and conversion, [E] Amino acid transport and metabolism, [F] Nucleotide transport and metabolism, [G] Carbohydrate transport and metabolism, [H] Coenzyme transport and metabolism, [I] Lipid transport and metabolism, [P] Inorganic ion transport and metabolism, [Q] Secondary metabolites biosynthesis, transport, and catabolism; POORLY CHARACTERIZED [R] General function prediction only, [S] Function unknown.

The draft genome for *Microtetraspora* sp. AC03309 (JADDIL000000000) has a size of almost 9.5 kb (9,434,728 bp) with a DNA G+C content of 71.0 mol%. The draft genome has a capacity for 8625 protein encoding genes as well as rRNAs [5S (2), 16S (2), 23S (3)] and 64 tRNAs. Subsystem coverage is 31% which contributes to a total of 461 subsystems out of 8625 CDS predicted by RAST server (Supplementary Figure S4). Predicted proteins were further analyzed into different functional classes based on Cluster of Orthologous Groups (COGs). Out of 7006 proteins, 4995 were mapped to unique COGs, which were further classified into metabolism (33.80%), cellular processes and signaling (16.16%), information storage and processing (20.77%) and poorly characterized (19.53%) functional classes. The remaining 9.75% proteins were assigned to more than one of the COGs categories and were grouped into Multiple COG (Figure A1, Appendix A). CRISPRFinder detected two confirmed CRISPR sites accessible for bacteriophage exposure and genome modification (Table A1, Appendix A).

- Specialty Genes:

Two antibiotic-resistant genes were predicted using CARD database (strict criteria), with functions in antibiotic resistance towards elfamycin (ARO: 3003361) and rapamycin antibiotic (ARO:3002883) (Table A2). A total of three drug targets were verified using DRUGBANK; 1 putative gene encoding Serine/threonine-protein kinase PknB, PknB [Pfam: Pkinase 379 (PF00069), PASTA (PF03793)], 1 putative gene encoding Recombinase A, RecA (Pfam: 380 PF00154), 1 putative gene encoding cell division protein FtsZ, ftsZ [Pfam: Tubulin 381 (PF00091) and FtsZ_C (PF12327] [Pfam: ProteasomePF00227] (Table A3).

- Secondary Metabolite Clusters:

A total of 26 secondary metabolite biosynthetic gene clusters (BGCs) were predicted using AntiSMASH software (Table A4). Four BGCs showed more than a 50% similarity to known compounds (geosmin, desferrioxamine E, alkylresorcinol and catenulipeptin), 13 BGCs with less than a 50% similarity to known compounds and 9 BGCs with no match. Interestingly, 2 BGCs of type I polyketide synthase (T1PKS) were detected, with BGCs of a 25% similarity to nostopeptolide A2 and a 40% similarity to labyrinthopeptin A2. T1PKS has been extensively studied and found in fungi, producing structurally diverse bioactive molecules, with very few found in bacteria [63].

- Sulfur Metabolism:

Desulfurization of vulcanized rubber is needed to reformulate and reuse rubber. Microorganism’s ability to break the sulfur bonds (C-S) in vulcanized rubber would provide higher surface area for latex-clearing protein (Lcp) to cleave the polymer backbone chain during biodegradation. *Microtetraspora* sp. AC03309 draft genome contained genes encoding for inorganic and organic sulfur metabolism (KEGG pathway ko:00920 and ko:02010). A total of 76 putative genes for sulfur metabolism were detected, with 21 genes categorized under general sulfur metabolism, 28 genes under inorganic sulfur metabolism and 27 genes under organic sulfur metabolism. Most of the genes encode organic sulfur metabolism of alkanesulfonate and inorganic sulfur assimilation of sulfate/thiosulfate metabolism (Table A5).

#### 3.4.4. Identification of Latex Clearing Protein (Lcp) Homolog for *Microtetraspora* sp. AC03309

**Figure 3:**
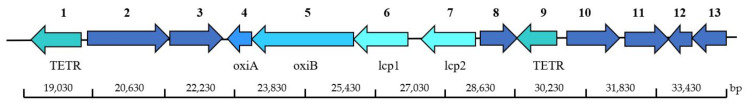
Location of *lcp1* and *lcp2*, and adjacent genes in *Microtetraspora* sp. AC03309 located on chromosome (contig 3): 1, Transcriptional regulator, TETR family; 2, Long chain fatty acid CoA ligase (EC 6.2.1.3); 3, Acetyl CoA dehydrogenase, short chain specific (EC 1.3.99.2); 4; Isoquinoline 1-oxidoreductase alpha subunit (*oxiA*); 5, Isoquinoline 1-oxidoreductase beta subunit (*oxiB*); 6, latex clearing protein 1 (*lcp1*); 7, latex clearing protein 2 (*lcp2*); 8, hypothetical protein; 9, Transcriptional regulator, TETR family; 10, MFS transporter; 11, hypothetical protein; 12, N-acetyltransferase; 13, pirin.

NCBI Conserved Domain Database (CDD) search, shows that *Microtetraspora* sp. AC03309 lcp-homologs (*lcp1* gene and *lcp2* gene) belongs to the DUF2236 domain-containing protein (accession pfam 09995) similar to latex-clearing protein (Lcp) for *Streptomyces* sp. K30. This is the first operon (lcp-oxiAB) structure reported for 2-lcp homolog located on the chromosome. *Microtetraspora* sp. AC03309 *lcp1* and *lcp2* genes were located adjacent to each other (Figure 3). Both lcp amino acid sequences showed a 58.50% similarity and contained the 13-residue long highly conserved region.

The *lcp1* gene (MW659698) is located on contig 3 at 26,812 to 25,649 bp. CDS translation results in 427 amino acids, encoding a protein with a theoretical mass of 47.0 kDa and 5.81 pI. Twin arginine translocation (TAT) signal peptide cleavage site was predicted between 30 and 31 bp (ARA-MP). Putative ribosomal site (RBS) was detected at 2 bp upstream from the putative start codon. The *lcp1* gene (1281 bp) showed 80.20% blast homology to latex clearing protein of *Actinophytocola xinjiangensis* (BLA60_08475). Lcp1 amino acid sequence was 51.37% similar to characterized Lcp of *Gordonia polyisoprenivorans* VH2 (ABV68923.1).

The *lcp2* gene (MW659699) is located on contig 3 at 28,435 to 27,206 bp, 277 bp upstream of *lcp1* gene. CDS translation results in 410 amino acids, encoding a protein with a theoretical mass of 44.8 kDa and 6.55 pI. A twin arginine translocation (TAT) signal peptide cleavage site was predicted between 30 and 31 bp (AQA-RT). A putative ribosomal site (RBS) was detected at 3 bp upstream from the putative start codon. The *lcp2* gene (1230 bp) showed 75.50% blast homology to latex-clearing protein of *Streptosporangium minutum* (A0A243RYB8_9ACTN). Lcp1 amino acid sequence had a 63.66% similarity to characterized Lcp of *Gordonia polyisoprenivorans* VH2 (ABV68923.1).

#### 3.4.5. Predicted Genes Involved in Rubber Degradation for *Microtetraspora* sp. AC03309

The degradation pathway of rubber was proposed by the identification of intermediate metabolites and experimental studies based on the genomes for *Streptomyces coelicolor* 1A, *Nocardia* sp. 835A, *Steroidobacter cummioxidans* sp. nov., strain 35Y, *Gordonia polyisoprenivorans* VH2 and *Nocardia nova* SH22a [12,23,64,65,66,67,68]. Based on these findings, putative genes that participate in the rubber degradation in *Microtetraspora* sp. AC03309 were identified in its draft genome (Table A6).

TetR-family is a regulatory mechanism that induces the production of Lcp protein during metabolization of rubber [69,70,71]. A total of 152 TETR-family genes were found. Two TETR encoding genes located near the *lcp* genes could be involved in the Lcp transcription (Figure 3). One TETR encoding gene is located 118 bp downstream of *lcp2* and *lcp1*, and another was located upstream 1023 bp of *lcp1* and *lcp2*. Lcp is secreted outside the bacterial cell via twin-arginine translocation (TAT) pathway. Four putative gene coding TAT proteins were identified. The *oxiB* (MZ726746) and *oxiA* (MZ726747) gene located downstream of *lcp1* and *lcp2* gene is an oxidoreductase that converts the oligo-isoprenoid terminal aldehyde group to a carboxylic acid group. Isoprenoid acids are then transported into the bacterial cell through Mammalian cell entry (Mce). Two candidate genes of MCE-family protein were found. Resulting isoprenoid acids enter the ß-oxidation cycle and are converted into acyl-CoA thioester by an acyl-CoA synthase (3 candidate genes). Acyl-CoA thioester are further catabolized by an acyl-CoA dehydrogenase (EC 1.3.8.1 & EC 1.3.8.7) (6 candidate genes). Polyunsaturated fatty acids degradation occur when 2,4-dienoyl-CoA reductase (EC 1.3.1.34) (1 candidate gene, NADH:flavin oxidoreductases, Old Yellow Enzyme family) catalyzes double bonds at the even-numbered position, followed by isomerization by enoyl-CoA hydratases (EC 4.2.1.17) (14 candidate genes). Six homologous genes for 3-hydroxyacyl-CoA dehydrogenases (EC 1.1.1.35) were identified, possibly involved in the following hydration step, responsible for the conversion of the hydroxyl derivatives into the keto. The last step of the first-oxidation cycle is predicted to be catalyzed by the thiolase/3-ketoacyl-CoA thiolase (EC 2.3.1.16) (6 candidate genes).

Mutants with a disruption of the α-methylacyl-CoA racemase (Mcr) gene lost the ability to metabolize poly-(*cis*-1,4-isoprene) and related methyl-branched isoprenoid compounds (Arenskötter et al., 2008). Three *mcr* genes (EC 5.1.99.4) were also identified in the genome of *Microtetraspora* sp. AC03309.

In addition, 2 candidate genes for superoxide dismutase (SodA)(EC 1.15.1.1) were also identified, believed to serve as a radical scavenger during degradation of poly(*cis*-1,4-isoprene), as the formation of SodA is induced during growth on rubber [72].

The presence of these genes in the genome of *Microtetraspora* sp. AC03309 suggests that this genome may share the same rubber degradation pathway as predicted previously.

### 3.5. Strain of Interest: Dactylosporangium sp. AC04546 (JCM 34239)

*Dactylosporangium* sp. AC04546 was isolated from a soil sample in a forest collected at Lanchang, Serian, Sarawak in 2007.

#### 3.5.1. Morphological Characterization of *Dactylosporangium* sp. AC04546

**Figure 4:**
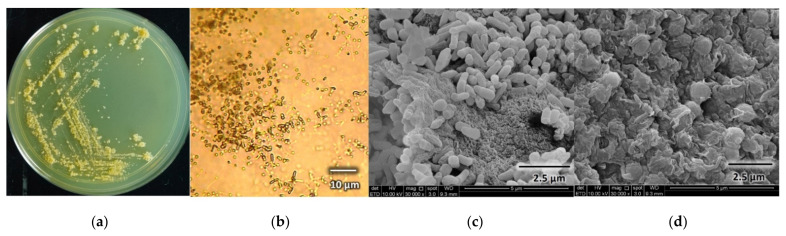
*Dactylosporangium* sp. AC04546 morphology: (**a**) surface morphology on Yeast Malt Extract (ISP2) agar; (**b**) spore-forming structures viewed using light microscope at 750× magnification on Soil Extract agar (SEA); (**c**) oblong-shaped sporangia viewed using scanning electron microscope (SEM Quanta FEG 650); (**d**) globose bodies viewed using scanning electron microscope (SEM Quanta FEG 650).

Morphological characteristics of *Dactylosporangium* sp. AC04546 can be seen in Figure 4. *Dactylosporangium* sp. AC04546 has yellowish orange surface and reverse color on ISP2 agar with moderate aerial mycelia. Colony is wrinkled with regular shape; it has an oblong shaped sporangia (~1.5 µm in length) with smooth surface, emerging directly from vegetative mycelium, arranged singly or in clusters. Globose bodies (~2.0 µm in diameter) were also observed. It is a mesophilic strain, growing well at 28 °C and up to 45 °C on ISP2 agar.

#### 3.5.2. *Dactylosporangium* sp. AC04546 Taxonomic Delineation

**Figure 5:**
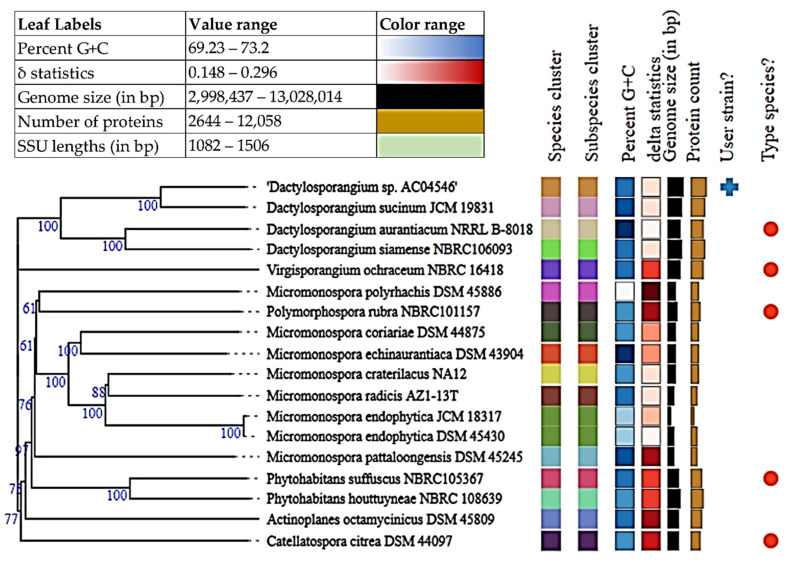
Tree inferred with FastME 2.1.6.1 (Lefort et al., 2015) from GBDP distances calculated from genome sequences. The branch lengths are scaled in terms of GBDP distance formula d5. The numbers above branches are GBDP pseudo-bootstrap support values > 60% from 100 replications, with an average branch support of 81.9%. The tree was rooted at the midpoint [62].

The complete 16S rRNA gene sequence for *Dactylosporangium* sp. AC04546 (1361 bp) was used for online BLASTN analysis against the 16S ribosomal RNA sequences (Bacteria and Archaea). *Dactylosporangium* sp. AC04546 had a 99.85% identity to *Dactylosporangium sucinum* strain RY35-23 (NR_145935.1) and genome-based phylogeny also shows that they are closely related (Figure 5) (TYGS, https://tygs.dsmz.de, accessed on 25 June 2021). Bacterial species are defined as including strains that present 95.00–96.00% of average nucleotide identity (ANI) and 70.00% of digital DDH (dDDH) [61]. Due to the low dDDH value of 44.40%, Average Amino Acid identity (AAI) was carried out [73]. Kostas lab server (http://enve-omics.ce.gatech.edu/g-matrix/, accessed on 27 June 2021) was used for this purpose. Amino acid sequences for *Dactylosporangium sucinum* JCM 19831 was obtained from NCBI Genome Assembly data (https://www.ncbi.nlm.nih.gov/assembly, accessed on 5 July 2021). AAI comparison resulted in a similarity of 89.64%. Accordingly, strains from the same microbial species share >95.00% Average Amino Acid Identity (AAI) [74].

ANI estimate of 91.63%, AAI of 89.64% and dDDH of 44.40% suggests that *Dactylosporangium* sp. AC04546 is a separate species from *Dactylosporangium sucinum*.

#### 3.5.3. *Dactylosporangium* sp. AC04546 Genomic Attributes

- Genomic & Protein Features

**Figure A2:**
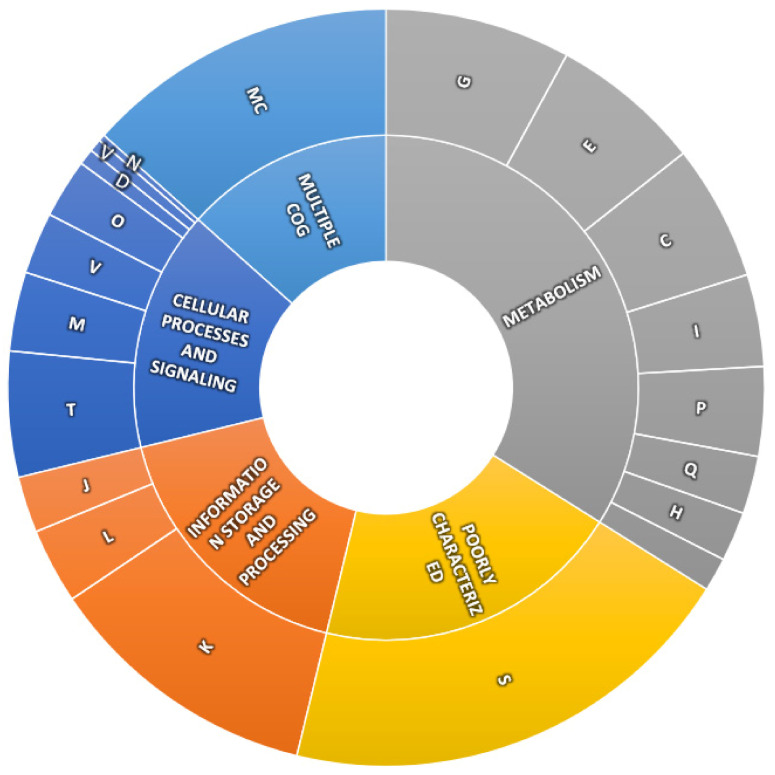
Cluster of Orthologous (COGs) for Dactylosporangium sp. AC04546 analyzed using egg-NOG mapper v.2 [42]. CELLULAR PROCESSES AND SIGNALING [D] Cell cycle control, cell division, chromosome partitioning, [M] Cell wall/membrane/envelope biogenesis, [N] Cell motility; [O] Post-translational modification, protein turnover, and chaperones, [T] Signal transduction mechanisms, [U] Intracellular trafficking, secretion, and vesicular transport, [V] Defense mechanisms [W] Extracellular structures, [Y] Nuclear structure, [Z] Cytoskeleton; INFORMATION STORAGE AND PROCESSING [A] RNA processing and modification, [B] Chromatin structure and dynamics, [J] Translation, ribosomal structure and biogenesis, [K] Transcription, [L] Replication, recombination and repair; METABOLISM [C] Energy production and conversion, [E] Amino acid transport and metabolism, [F] Nucleotide transport and metabolism, [G] Carbohydrate transport and metabolism, [H] Coenzyme transport and metabolism, [I] Lipid transport and metabolism, [P] Inorganic ion transport and metabolism, [Q] Secondary metabolites biosynthesis, transport, and catabolism; POORLY CHARACTERIZED [R] General function prediction only, [S] Function unknown.

The draft genome for *Dactylosporangium* sp. AC04546 (JAHYSG000000000) has a size of approximately 13 kb (13,028,014) with a DNA G+C content of 72.1 mol%. The draft genome has a capacity for 12,556 protein encoding genes as well as rRNAs [5S (1), 16S (4), 23S (3)] and 88 tRNAs. *Dactylosporangium* sp. AC04546 draft genome consists of 16% subsystem coverage which contributes to a total of 334 subsystems out of 12,556 CDS predicted by the RAST server (Supplementary Figure S5 Predicted proteins were further analyzed into different functional classes using Cluster of Orthologous Groups (COGs) (Figure A2, Appendix B). Out of 7006 proteins, 4995 were mapped to unique COGs which were further classified into metabolism (33.93%), cellular processes and signaling (32.82%), information storage and processing (17.40%) and poorly characterized (19.86%) functional classes. The remaining 13.39% proteins were assigned to more than one COGs category and were grouped into Multiple COG. A total of 11 confirmed CRISPR loci, indicates the high ability of *Dactylosporangium* sp. AC04546 naturally occurring genome editing system which serves as genome editing sites (Table A7, Appendix B).

- Specialty Gene:

Antibiotic-resistant genes, 2 genes (strict criteria) with functions in antibiotic resistance towards aminoglycoside (ARO:3002644) and glycopeptide antibiotic (ARO:3005036) were detected (Table A8). Two drug targets were detected using PATRIC 539 server and verified using DRUGBANK, 1 putative gene encoding GTP binding (Pfam: 540 Tubulin (Pfam: PF00091) and FtsZ_C (Pfam: PF12327) and 1 putative gene encoding re- 541 combinase A, Rec A (Pfam: PF00154) (Table A9).

- Secondary metabolite clusters:

AntiSMASH software predicted 19 secondary metabolite biosynthetic gene clusters (BGCs) (Table A10). Two BGCs showed more than 50% similarity to arenimycin (68%) and alkyl-O-dihydrogeranyl-methoxyhydroquinones (57%), respectively, 10 BGCs below 50%, and 7 BGCs with no similarity.

- Urea Metabolism:

Based on the draft genome of *Dactylosporangium* sp. AC04546, it is predicted to be able to utilize urea as the sole nitrogen source during N-limiting conditions. Presence of putative genes for urea transporters (*urtA, urtB, urtC, urtD, urtE*) and urease operon (*ureA, ureB, ureC, ureD, ureE, ureF, ureG*) were identified [75] (Table A11). The potential of using *Dactylosporangium* sp. AC04546 strain or enzyme for bioremediation expands beyond rubber biodegradation.

- Polyhydroxybutyrate Metabolism

**Figure A3:**
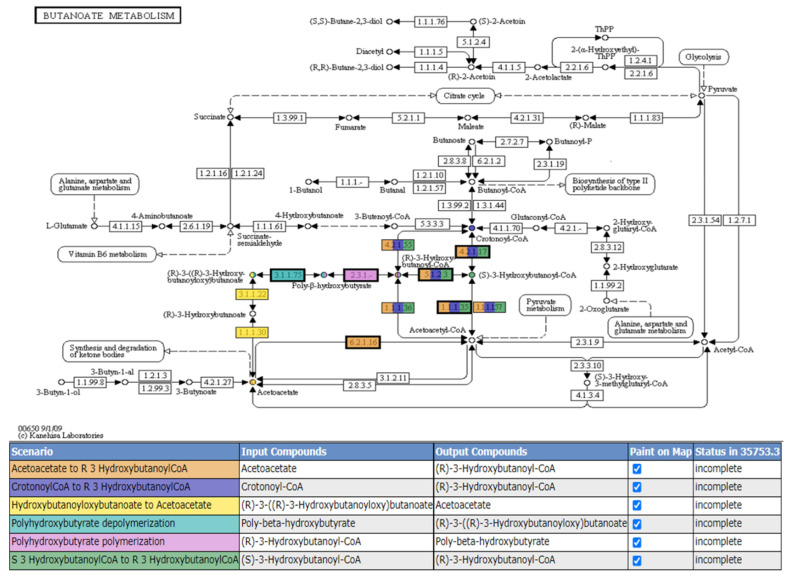
*Dactylosporangium* sp. AC04546 polyhydroxybutyrate metabolism subsystem based on KEGG pathway, predicted by RAST server, and viewed using The SEED Viewer. Color on the MAP corresponds to the predicted scenario in the legend below.

A total of 88 putative genes involved in polyhydroxybutyrate metabolism were found. Six scenarios were predicted by The SEED Viewer but were incomplete (Figure A3). Poly-3-hydroxybutyrate (PHB) is a biological polyester present in bacteria and eukaryotic cells stored for carbon and energy storage during stress, and is extensively studied for production of biodegradable plastics [76].

#### 3.5.4. Identification of Latex Clearing Protein (Lcp) Homolog for *Dactylosporangium* sp. AC04546

NCBI Conserved Domain Database (CDD) search, shows that *Dactylosporangium* sp. AC04546 lcp-homologs (*lcp1*, *lcp2* and *lcp3* gene) belong to the DUF2236 domain-containing protein (accession pfam 09995). All the lcp-homologs contains the 13-residue long conserved region.

**Figure 6:**
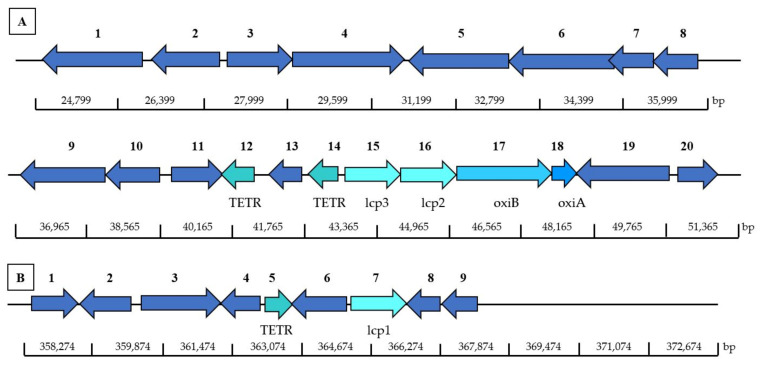
Location of *lcp1*, *lcp2* and *lcp3*, and adjacent genes in *Dactylosporangium* sp. AC04546 located on chromosome: (**A**) Contig 82: 1, Long chain fatty acid CoA ligase (EC 6.2.1.3); 2, Transcriptional regulator, AcrR; 3, Acetyl-CoA Acetyltransferase (EC 2.3.1.9); 4, Enoyl-CoA Hydratase [isoleucine degradation] (EC 4.2.1.17)/3-Hydroxyl-CoA dehydrogenase (EC 1.1.135)/3-Hydroxyl-CoA butyryl CoA epimerase (EC 5.1.2.3); 5, Long chain fatty acid CoA ligase (EC 6.2.1.3); 6, Oxidoreductase, GMC family; 7, ABC transporter; 8, branch chain amino acid transport ATP-binding protein LivG; 9, High affinity branched chain amino acid transport system permease protein LivH (TC 3.A.1.4.1); 10, ABC transporter substrate binding protein; 11, Acyl-CoA dehydrogenase; 12, Transcriptional regulator, TETR family; 13, oxidoreductase, short chain dehydrogenase/reductase family; 14, Transcriptional regulator, TETR family; 15, latex clearing protein gene 3 (*lcp3*); 16, latex clearing protein gene 2 (*lcp2*); 17, Isoquinoline 1-oxidoreductase beta subunit (*oxiB*); 18, Isoquinoline 1-oxidoreductase alpha subunit (*oxiA*), 19, GGDEF domain containing protein; 20, RNA polymerase sigma-70 factor. (**B**) Contig 1: 1, S8 family serine peptidase; 2, hypothetical protein; 3, two component system sensor histidine kinase; 4, NmrA family protein; 5, Transcriptional regulator, TETR family; 6, Transcriptional regulator, AcrR family; 7, latex clearing protein gene 1 (*lcp1*); 8, HTH-type transcriptional activator TipA; 9, two component system sensor histidine kinase.

The operon structure (lcp-oxiAB) of *Dactylosporangium* sp. AC04546 is similar to previously reported strains containing 3-lcp homologs; *Streptomyces* sp. CFMR-7 [77] and *Actinoplanes* sp. strain OR16 [78], whereby 2-lcp homologs are located adjacent to each other, followed by *oxi*AB genes, with 1-lcp homolog located far apart (Figure 6). The *lcp2* and *lcp3* gene for *Dactylosporangium* sp. AC04546 were located adjacent to each other on contig 82, while *lcp1* gene was located on contig 1 (Figure 6). Amino acid sequences of Lcp2 and Lcp3 are 60.8% similar. Amino acid sequences for Lcp1 and Lcp2 have a similarity of 48.3% while Lcp1 and Lcp3 have a 45.3% similarity.

The *lcp1* gene (MW659700) is located on contig 1 from 364,865 to 366,085 bp. CDS translation results in 407 amino acids, encoding a protein with a theoretical mass of 44.2 kDa and 9.00 pI. Twin arginine translocation (TAT) signal peptide cleavage site was predicted to be between 40 and 41 bp (ALA-AP). Putative ribosomal site (RBS) was detected at 6 bp upstream from the putative start codon. The *lcp1* gene (1221 bp) showed 80.70% blast homology to latex clearing protein of *Amycolatopsis* sp. YIM 10 (A0A5P9PKU1_9PSEU). Lcp1 amino acid sequence a 40.70% similarity to characterized Lcp of *Gordonia polyisoprenivorans* VH2.

The *lcp2* gene (MW659701) is located on contig 82 from 43,562 to 44,770 bp. CDS translation results in 403 amino acids, encoding a protein with a theoretical mass of 43.8 kDa and 6.33 pI. Twin arginine translocation (TAT) signal peptide cleavage site was predicted to be between 40 and 41 bp (AGA-GA). Putative ribosomal site (RBS) was detected at 9 bp upstream from the putative start codon ATG. The *lcp2* gene (1200 bp) showed 87.60 % blast homology to latex clearing protein of *Nonomuraea* sp. ATCC 55076 (A0A1U9ZY53_9ACTN). Lcp1 amino acid sequence had a 65.33% similarity to Lcp of *Gordonia polyisoprenivorans* VH2 characterized.

The *lcp3* gene (MW659702) is located on contig 82 from 44,862 to 46,061 bp. CDS translation results in 400 amino acids, encoding a protein with a theoretical mass of 43.9 kDa and 5.93 pI. Twin arginine translocation (TAT) signal peptide cleavage site was predicted between 40 and 41 bp (AGA-GA). Putative ribosomal site (RBS) was detected at 9 bp upstream from the putative start codon ATG. The *lcp3* gene (1224 bp) showed 84.00% blast homology to latex clearing protein of *Actinophytocola xinjiangensis* (A0A7Z0WP15_9PSEU). Lcp1 amino acid sequence had a 50.63% similarity to characterized Lcp of *Gordonia polyisoprenivorans* VH2 (ABV68923.1).

#### 3.5.5. Predicted Genes Involved in Rubber Degradation for *Dactylosporangium* sp. AC04546

Putative genes that may participate in the rubber degradation in *Dactylosporangium* sp. AC04546 were identified in its draft genome (Table A12). Five putative TER-family gene were identified, 2 genes located 119 bp and 2218 bp downstream of the *lcp2, lcp3*. One gene belonging to the TETR-family, located 1431 bp downstream *lcp1* gene, could be involved in the Lcp transcription (Figure 6). Lcp are secreted outside the bacterial cell via twin-arginine translocation (TAT) pathway. Four putative gene-coding TAT proteins (*TatA, TatB, TatC*) were identified. The *oxiAB* gene, located downstream of the *lcp3* and *lcp2* gene, is an oxidoreductase that converts the oligo-isoprenoid terminal aldehyde group to a carboxylic acid group. Oligo-isoprenoids are then transported into the bacterial cell. No specific MCE-family genes were detected. The resulting isoprenoid acids enter the ß-oxidation cycle and are converted into acyl-CoA thioester by an acyl-CoA synthase (2 candidate gene). Acyl-CoA thioester are further catabolized by an acyl-CoA dehydrogenase (EC 1.3.8.1 & EC 1.3.8.8) (6 candidate genes). Polyunsaturated fatty acids degradation occurs when 2,4-dienoyl-CoA reductase (EC 1.3.1.34) (1 candidate genes) catalyzes double bonds at the even-numbered position, followed by isomerization by enoyl-CoA hydratases (EC 4.2.1.17) (14 candidate genes). Fourteen homologous genes for 3-hydroxyacyl-CoA dehydrogenases (EC 1.1.1.35) were identified, possibly involved in the following hydration step, responsible for the conversion of the hydroxyl derivatives into the keto. The last step of the first-oxidation cycle is predicted to be catalyzed by the thiolase/3-ketoacyl-CoA thiolase (EC 2.3.1.16) (15 candidate genes).

Mutants with a disruption of the α-methylacyl-CoA racemase (*Mcr*) gene lost the ability to metabolize poly-(*cis*-1,4-isoprene) and related methyl-branched isoprenoid compounds (Arenskötter et al., 2008). Three *Mcr* genes (EC 5.1.99.4) were also identified in the genome of *Dactylosporangium* sp. AC04546.

The presence of these genes in the genome of *Dactylosporangium* sp. AC04546 suggests that this genome may share the same rubber degradation pathway as predicted previously.

### 3.6. Utilization of Rubber Materials by Microtetraspora sp. AC03309 and Dactylosporangium sp. AC04546

When cultivated in an ISP2 broth at 28 °C, 180 rpm, *Microtetraspora* sp. AC03309 was actively growing by Day 7 while *Dactylosporangium* sp. AC04546 by Day 10. Actively growing culture was used to inoculate Mineral Salts Medium (MSM) containing rubber as the sole carbon source. Rubber utilization studies were conducted separately for both strains.

**Figure 7:**
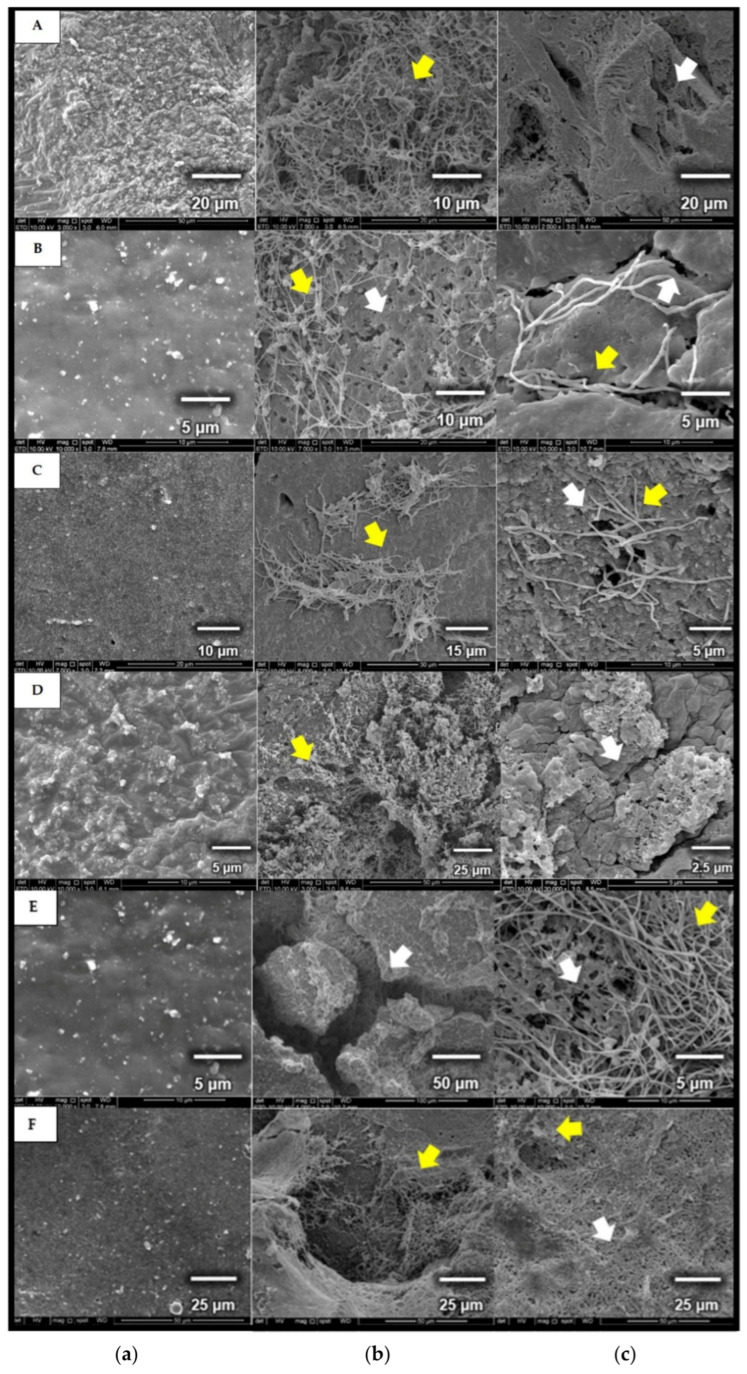
Micrographs of (**a**) control, rubber samples without microbial cultivation (A & D: fresh latex pieces; B & E: latex glove pieces; C & F: tyre granules); (**b**,**c**) rubber samples used as carbon source for cultivating *Microtetraspora* sp. AC03309 (A,B,C) and *Dactylosporangium* sp. AC04546 (D,E,F), incubated for 30 days, 28 °C at 180 rpm.

After 30 days of incubation, SEM images of rubber materials showed that the strains were able to grow and utilize fresh latex, latex glove, or tyres as the sole carbon and energy source. The biodegradation of the rubber polymer begins with microbial attachment on the surface, which has thread-like appearances (yellow arrow) in comparison to non-inoculated samples [Figure 7]. Once attached, the microorganism releases degrading enzymes through its mycelia, initiating the first step of rubber degradation. This can be seen through the presence of rough, cracked, and holes (white arrow) on the rubber materials [Figure 7b,c].

### 3.7. ATR-FTIR Analysis of Degraded Rubber Materials

ATR-FTIR spectroscopy is a useful tool to determine the formation or disappearance of functional groups of materials that indicate degradation of the original material. The infrared spectroscopy (IR) can be divided into 4 categories: (i) single bond area (4000 cm^−1^–2500 cm^−1^), (ii) triple bond region (2500 cm^−1^–2000 cm^−1^), (iii) double bond region (2000 cm^−1^–1500 cm^−1^) and (iv) fingerprint region (1500 cm^−1^–600 cm^−1^) [79]. During rubber utilization, Lcp catalyzes the oxidative C-C cleavage of poly(*cis*-1,4-isoprene) in natural rubber as well as in synthetic rubber by the addition of oxygen (O_2_) to the double bonds, leading to a mixture of oligonucleotide-isoprenoids with terminal keto and aldehyde groups (endo-type cleavage) [9,11,80].

#### 3.7.1. Fresh Latex ATR-FTIR Profile

**Figure 8:**
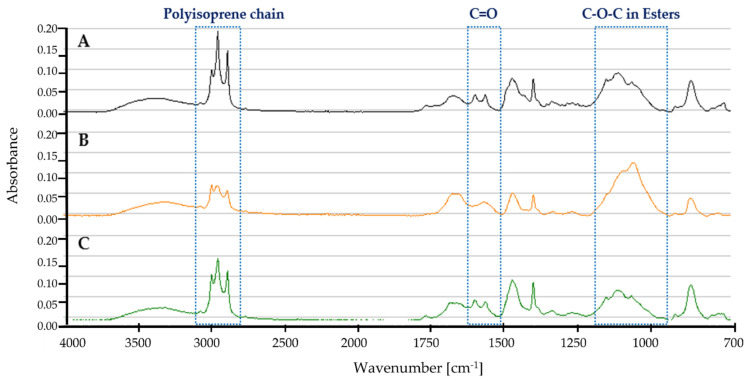
ATR-FTIR profile for fresh latex pieces as carbon source in MSM broth for 30 days, 28 °C, 180 rpm (**A**) Control (**B**) *Dactylosporangium* sp. AC04546 (**C**) *Microtetraspora* sp. AC03309.

Figure 8 shows the ATR-FTIR profile for fresh latex (A) control, no microbial culture added, (B) cultivated with *Dactylosporangium* sp. AC04546, and (C) cultivated with *Microtetraspora* sp. AC03309.

Characteristic bands of the polyisoprene chain at 2962 cm^−1^, 2928 cm^−1^ and 2855 cm^−1^ were present [81]. Decreased intensity of this spectrum for *Dactylosporangium* sp. AC04546 and *Microtetraspora* sp. AC03309 indicates the degradation of fresh latex samples. Degradation of fresh latex should result in the appearance of hydroxyl, carbonyl (aldehyde, ketone, and/or carboxylic acid) and ester groups [81]. A range of between 1750 cm^−1^ and 1700 cm^−1^ describes simple carbonyl compounds (ketones, aldehydes, esters, or carboxyl). Peak below 1700 cm^−1^ corresponds to carbonyl with amides or carboxylates functional group, while intensity at between 1650 cm^−1^ and 1600 cm^−1^ is due to the presence of double bonds. Strong peak profile at 1200 cm^−1^–1000 cm^−1^ for *Dactylosporangium* sp. AC04546 is caused by C-O-C stretching vibration in esters. Similar changes of profile was also reported in natural rubber after 1 year aging at temperate temperature compared to control [82].

#### 3.7.2. Latex Glove ATR-FTIR Profile

**Figure 9:**
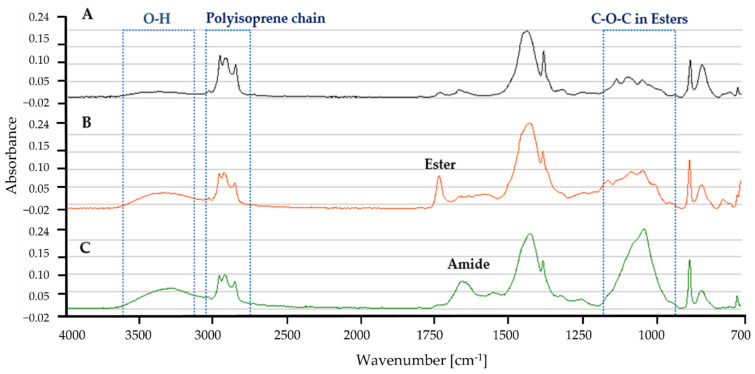
ATR-FTIR profile for latex glove strips as carbon source in MSM broth for 30 days, 28 °C, 180 rpm (**A**) Control (**B**) *Dactylosporangium* sp. AC04546 (**C**) *Microtetraspora* sp. AC03309.

Figure 9 shows the ATR-FTIR profile for latex gloves in MSM with (A) control, no microbial culture added, (B) *Dactylosporangium* sp. AC04546, and (C) *Microtetraspora* sp. AC03309. Characteristic bands of the polyisoprene chain at 2962 cm^−1^, 2928 cm^−1^ and 2855 cm^−1^ [81] were also present in the latex glove profiles. A broad absorption band in the range of 3650 and 3250 cm^−1^ for *Dactylosporangium* sp. AC04546 and *Microtetraspora* sp. AC03309, is caused by O-H stretching vibrations in the carboxylic groups, as a result of rubber degradation by oxidation. At 1750 cm^−1^–1725 cm^−1^ a distinct peak for ester was detected in the profile for *Dactylosporangium* sp. AC04546. While a distinct peak at 1680–1630 cm^−1^ for amide appeared in the IR for *Microtetraspora* sp. AC03309, obvious change in the peak profile at 1200–1000 cm^−1^ was caused by C-O-C stretching vibration in esters.

#### 3.7.3. Tyre ATR-FTIR Profile

**Figure 10:**
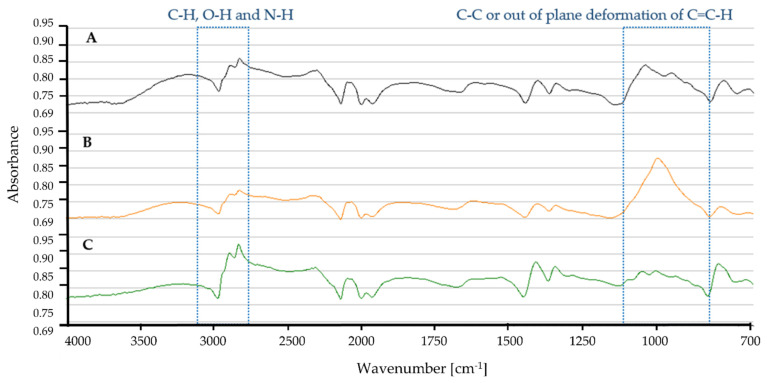
ATR-FTIR profile for tyre granules as carbon source in MSM broth for 30 days, 28 °C, 180 rpm (**A**) Control (**B**) *Dactylosporangium* sp. AC04546 (**C**) *Microtetraspora* sp. AC03309.

Based on the IR spectra, tyre samples incubated with *Dactylosporangium* sp. AC04546 showed differences at 3000 cm^−1^ and ~1000 cm^−1^ (Figure 10). The broadening of peaks at ~3000 cm^−1^ is caused by C-H, O-H and N-H stretching, while peak intensity at ~1000 cm^−1^ is due to stretching vibrations of the C-C and/out of plane deformation of C=C-H [83].

## 4. Discussion

We were able to quickly screen a portion of SBC Actinobacterial Culture Collection by modifying the screening method using natural rubber (NR) latex agar in 6-well plates instead of latex agar overlay technique as described by Braaz et al., (2004) [28]. We also prolonged the incubation period from 1 week to 4 weeks as some Actinobacteria strains (rare group) are known to be slow growers. Using 6-well plates, we prepared NR latex agar (without overlay) in smaller volume while utilizing minimal space. The separation of media by wells also avoids cross contamination, especially by sporulating strains. A similar method using NR latex agar in 24-well plates was not successful in detecting the formation of clear zones. Using MSM broth added with NR latex in test tubes, 6-well plate and 24-well plate did not show changes in the turbidity of the medium.

Back in 1997, a publication indicated that *Microtetraspora* sp. strain 3880-19B isolated from a soil sample collected in Malaysia, and *Dactylosporangium thailandense* DSM 43158 isolated from Thailand produced clearing zone on NR latex overlay agar [21]. However, no further studies on these strains have been reported. No molecular information or strain deposition was found for *Microtetraspora* sp. strain 3880-19B. When we compared the genome of *Microtetraspora* sp. AC03309 and *Microtetraspora glauca* strain NBRC 14761 using The SEED Viewer server, *Microtetraspora glauca* strain NRBC 14761 did not contain genes of Lcp-homolog, isoquinoline 1-oxidoreductase beta subunit (oxiB), isoquinoline 1-oxidoreductase alpha subunit (oxiA) and MCE-family protein. The genome search of *Microtetraspora glauca* using the GenBank database also confirms this. Only 1 genome for *Microtetraspora glauca* has been deposited to the NCBI Genome database. Although in silico taxonomic delineation classifies *Microtetraspora* sp. AC03309 as a novel subspecies under *Microtetraspora glauca*, based on the rubber-degrading ability and related rubber degradation gene content, we propose *Microtetraspora* sp. AC03309 to be considered as a novel species. In this study, we have successfully identified 7 *Microtetraspora* strains containing Lcp-homolog. It would be interesting also to look at the interspecies variation.

Comparison between 16S rDNA sequence of *Dactylosporangium* sp. AC04546 and *Dactylosporangium thailandense* DSM 43158 showed a similarity of 98.00%, which is below the recommended threshold of >98.65% 16S rRNA gene sequence similarity for differentiating two species [60]. Therefore, we believe both strains to be distinct. We then compared the genome of *Dactylosporangium* sp. AC04546 *to Dactylosporangium sucinum* JCM 19831. Interestingly, we found 3-Lcp homologs, putative *oxiB* and putative *oxiA* genes within the *Dactylosporangium sucinum* JCM 19831 genome. The *lcp3* gene and the *lcp2* gene were located on contig 30 together with *oxiAB*, while *lcp1* was located on contig 19 of *Dactylosporangium sucinum* JCM 19831. When we compared the Lcp-homologs amino acid sequence, Lcp1 of both sequences had a 95.07% similarity, Lcp2 had a 93.03% similarity and Lcp3 had a 97.24% similarity. Four MCE-family proteins were detected in the draft genome of *Dactylosporangium sucinum* JCM 19831 (genome size 12,103,263 bp) and was compared to the genome of *Dactylosporangium* sp. AC04546 (genome size 13,028,014 bp). No blast homology was detected. Therefore, further analysis of the *Dactylosporangium* sp. AC04546 membrane transporter proteins involved in the rubber degradation pathway are needed. Low ANI and dDDH values for *Dactylosporangium* sp. AC04546 *to Dactylosporangium sucinum* JCM 19831 suggest that they have distinct genomic differences as a separate species, while the full length Lcp amino acid comparison did not match 100.00%.

Almost all rubber-degrading Actinobacteria, including *Streptomyces*, *Nocardia*, and *Rhodococcus* species have a single Lcp homolog [78]. We predict that Actinobacterial strains adapt by incorporating lcp genes into their chromosome through their plasmid (e.g., *Gordonia polyisoprenivorans* VH2, 1 *lcp* gene in plasmid and in 1 *lcp* gene in its chromosome). Actinobacteria have been known to adapt quickly through incorporation of genes (horizontal gene transfer, HGT). The question is whether the incorporation of Lcp-homologs impact the ability of the strain to utilize and degrade different rubber products? Previous reports indicate that different Lcp produce a variety of rubber degraded products (molecular weight, number of isoprene units, functional groups, etc) [65]. This could most likely be due to the different or synergistic mechanism of Lcp, which is yet to be explored. Lcp genes from *Microtetraspora* sp. AC03309 and *Dactylosporangium* sp. AC04546 also did not cluster with other biochemically characterized Lcp (Supplementary Figure S6).

Dissimilar to other rubber products, tyres are made from vulcanized rubber and ~30% carbon black for reinforcement, rendering them more resistant to degradation. Vulcanization of rubber material through sulfur interlinkages of the poly(*cis*-1,4-isoprene) chains results in reduced water absorption and gas permeability of the material [65]. Based on the rubber utilization studies, we observed that both strains were able to colonize and utilize various rubber products differently. An obvious observation was the ability of *Dactylosporangium* sp. AC04546 to colonize the tyre samples effectively through excretion of rubber-degrading enzymes (appearance of holes on the tyre surface compared to control and samples incubated with *Microtetraspora* sp. AC03309). This is supported by the changes detected in the ATR-FTIR profile. Since tyres are the second largest contributor to microplastic pollution in the ocean [84], it would be interesting to study the potential of *Dactylosporangium* sp. AC04546 in degrading tyre wastes.

Most of the clear zone producing strains (61%) were isolated from soil samples collected from Kiding Village Forest area. The Kiding village settlement was founded in the 1840s and is located 1300 m above sea level; it is only accessible by a 3-h hike. While all the other NR latex degrading strains were isolated from secondary forest soil samples, the isolation sites for all the strains have no obvious rubber wastes or rubber materials present, so there does not appear to be an apparent evolutionary pressure for the appearance of lcp genes. Nonetheless, these strains do degrade rubber, and it is possible that this function may have been triggered by the presence of rubber particles dispersed in the environment. Recent studies have shown that rubber particles from sources such as tyres are widely dispersed. Sieber et al., (2020), estimated that 218 ktons of rubber particles from tyres are mainly deposited on road-side soils (74%), surface water (22%) and in soils (4%) [85]. Alternatively, the presence of latex producing plants in the vicinity may have contributed to the development of Lcp in these strains. Further studies would be required to confirm the reasons for this. But this observation highlights the importance of surveying microorganisms from diverse ecosystems (e.g., marine, or freshwater bodies) for their genomic background and functional ability related to biodegradation of rubber or other pollutants. 

## 5. Conclusions

Miniaturized random screening using Sarawak’s Actinobacterial Culture Collection led to the successful identification of 18 natural rubber latex degrading Actinobacteria isolated from environmental soil samples. Two rare Actinobacteria strains with uncharacterized lcp genes, *Microtetraspora* sp. AC03309 and *Dactylosporangium* sp. AC04546 were explored. *Microtetraspora* sp. AC03309 a proposed novel species has 2 Lcp homologs on its chromosome, while *Dactylosporangium* sp. AC04546 is a proposed novel species that has 3 Lcp homologs on its chromosome. The identification of genes predicted to be involved in the rubber-degradation pathway was established for both strains and corresponds to the genes identified in other rubber-degrading Actinobacteria. The presence of other assimilation genes such as sulfur in *Microtetraspora* sp. AC03309, and urea and polyhydroxybutyrate in *Dactylosporangium* sp. AC04546 provides them with added advantages as biodegraders. Both strains were also able to colonize and utilize rubber-based materials within 30 days of incubation. Interestingly, *Dactylosporangium* sp. AC04546 grew well in the presence of tyre samples as the sole carbon and energy source, showing visible changes on the tyre surface seen in SEM images and changes in the ATR-FTIR spectra within 30 days of incubation. Further studies on the ability of this strain (or a combination of strains) to effectively degrade tyre products and detailed analysis of the degraded rubber products would be of interest for future research.

## Figures and Tables

**Table 1:** Screening of Sarawak Biodiversity Centre (SBC) Actinobacteria Culture Collection for rubber degraders.

Family	Genera	No. of Strains Tested	Clear Zone Formation
Streptomycetaceae	*Streptomyces*	526	2
	*Kitasatospora*	12	0
Micromonosporaceae	*Micromonospora*	86	6
	*Dactylosporangium*	3	1
Streptosporangiaceae	*Nonomuraea*	31	2
	*Microtetraspora*	11	7
	*Microbispora*	4	0
	*Herbidospora*	2	0
Thermoactinomycetaceae	*Shimazuella*	1	0
Thermomonosporaceae	*Actinomadura*	8	0
	*Actinoallomurus*	1	0
Pseudonocardiaceae	*Actinomycetospora*	1	0
Thermosporotrichaceae	*Thermosporothrix*	1	0
Mycobacteriaceae	*Mycobacterium*	2	0
Nocardiaceae	*Nocardia*	46	0
	Others	205	0
		940	18

Identification of the strains were based on the strain’s morphology, spore-forming structures, some having 16S rRNA molecular information. Others: unable to determine genus based on morphology.

## Data Availability

Contigs used in this study were deposited into the National Centre for Biotechnology Information (NCBI) database; *Microtetraspora* sp. AC03309 under the BioProject ID: PRJNA668755, with BioSample Accessions SUB8307686 and *Dactylosporangium* sp. ACO4546 under the BioProject ID: PRJNA749440, with BioSample Accessions SUB10069292. All the other data supporting the findings of this study are available in this published article and its supplementary information files.

## Supplementary Materials

The following are available online at https://www.mdpi.com/article/10.3390/polym13203524/s1, Figure S1: Research approach flow chart; Figure S2: Clear zone forming strains 16S rRNA evolutionary relationships of taxa together with top 1 blast homology in GenBank database; Figure S3: Latex clearing protein (Lcp) amino acid sequence alignment of the 18 clear zone strains to determine the 13-residue long highly conserved region; Figure S4: Subsystem distribution in *Microtetraspora* sp. AC03309; Figure S5: Subsystem distribution in *Dactylosporangium* sp. AC04546; Figure S6: Phylogenetic tree of characterized Lcps and Lcps from *Microtetraspora* sp. AC03309 and *Dactylosporangium* sp. AC04546.

## Appendix A

**Table A1:** *Microtetraspora* sp. AC03309 CRISPR array detected from CRISPRFinder server.

	Contig	Start (bp)	Stop (bp)
1	contig000014	94,175	94,462
2	contig000145	766	977

**Table A2:** *Microtetraspora* sp. AC03309 antibiotic resistant gene identified using CARD database.

	Contig	Start (bp)	Stop (bp)	Classification	Identity (%)
1	contig000052	61,507	62,700	elfamycin (ARO: 3003361)	77
2	contig 000084	35,081	36,280	rapamycin antibiotic (ARO:3002883)	93

**Table A3:** *Microtetraspora* sp. AC03309 drug target gene identified using PATRIC server and DrugBank server.

	Contig	Product	Start (bp)	Stop (bp)	Length (bp)	Query Coverage	Identity (%)
1	Contig000007	RecA protein	27,312	25,081	1104	88	85
2	Contig000641	Serine/threonine-protein kina, pknB (EC 2.7.11.1)	35	322	288	90	88
3	Contig000054	Cell division protein, ftsZ	15,100	13,667	1434	95	92

**Table A4:** *Microtetraspora* sp. AC03309 putative genes involved in sulfur assimilation annotated using RAST server.

Roles		ORF
**Inorganic sulfur: Sulfate/thiosulfate metabolism**		
1	3’(2’),5’-bisphosphate nucleotidase (EC 3.1.3.7)	1
2	Adenylylsulfate kinase (EC 2.7.1.25)	2
3	Adenylyl-sulfate reductase [thioredoxin] (EC 1.8.4.10)	3
4	Ferredoxin	12
5	Ferredoxin-like protein involved in electron transfer	1
6	Ferredoxin--NADP(+) reductase, actinobacterial (eukaryote-like) type (EC 1.18.1.2)	1
7	Ferredoxin--sulfite reductase, actinobacterial type (EC 1.8.7.1)	1
8	Phosphoadenylyl-sulfate reductase [thioredoxin] (EC 1.8.4.8)	1
9	Putative sulfate permease	1
10	Sulfate adenylyltransferase subunit 1 (EC 2.7.7.4)	1
11	Sulfate adenylyltransferase subunit 2 (EC 2.7.7.4)	1
12	Sulfate adenylyltransferase, dissimilatory-type (EC 2.7.7.4)	1
13	Sulfate and thiosulfate binding protein CysP	1
14	Sulfate and thiosulfate import ATP-binding protein CysA (EC 3.6.3.25)	2
15	Sulfate transport system permease protein CysW	1
**Organic sulfur: Alkanesulfonate assimilation**		
1	ABC-type nitrate/sulfonate/bicarbonate transport system, ATPase component	1
2	ABC-type nitrate/sulfonate/bicarbonate transport system, permease component	2
3	ABC-type nitrate/sulfonate/bicarbonate transport systems, periplasmic components	1
4	Alkanesulfonate monooxygenase (EC 1.14.14.5)	2
5	Alkanesulfonates ABC transporter ATP-binding protein	1
6	Alkanesulfonates transport system permease protein	3
7	Alkanesulfonates-binding protein	1
8	Alpha-ketoglutarate-dependent taurine dioxygenase (EC 1.14.11.17)	1
9	Arylsulfatase (EC 3.1.6.1)	1
10	FMN reductase (EC 1.5.1.29)	1
11	Putative arylsulfatase regulatory protein	1

ORF: Open reading frame.

**Table A5:** *Microtetraspora* sp. AC03309 secondary metabolite biosynthetic gene clusters (BGCs) were predicted using AntiSMASH 6.0.

Region	Type	From (bp)	To (bp)	Most Similar Known Cluster		Similarity
Region 5.1	other	406	41,026			
Region 7.1	NRPS	58,409	104,012	tetronasin	Polyketide	3%
Region 8.1	redox-cofactor	1	16,071			
Region 8.2	lanthipeptide-class-iv	119,417	142,221			
Region 11.1	lanthipeptide-class-iv	4363	27,032	blasticidin S	Other	7%
Region 11.2	NRPS,T1PKS	87,888	145,746	nostopeptolide A2	Polyketide + NRP:Cyclic depsipeptide	25%
Region 14.1	T3PKS	105,469	141,262	alkylresorcinol	Polyketide	66%
Region 16.1	RiPP-like	121,803	132,618			
Region 17.1	terpene	20,289	46,240	hopene	Terpene	46%
Region 26.1	terpene	88,473	108,659	frankiamicin	Polyketide	14%
Region 28.1	NRPS	1	28,384	5-acetyl-5,10-dihydrophenazine-1-carboxylic acid/5-(2-hydroxyacetyl)-5,10-dihydrophenazine-1-carboxylic acid/endophenazine A1/endophenazine F/endophenazine G	Other:Phenazine	8%
Region 34.1	betalactone,terpene	62,313	94,083	geosmin	Terpene	100%
Region 39.1	lanthipeptide-class-iii	66,288	83,988			
Region 44.1	NRPS	1	42,513	acarviostatin I03/acarviostatin II03/acarviostatin III03/acarviostatin IV03	Saccharide	11%
Region 44.2	T1PKS,lanthipeptide-class-iv	48,145	75,803	labyrinthopeptin A2/labyrinthopeptin A1/labyrinthopeptin A3	RiPP:Lanthipeptide	40%
Region 45.1	terpene	24,504	45,502			
Region 49.1	NRPS	1	32,408	herboxidiene	Polyketide	3%
Region 57.1	thiopeptide,LAP	33,174	64,696	muraymycin C1	NRP + Polyketide	7%
Region 61.1	siderophore	17,145	30,431			
Region 67.1	NRPS	1	50,370	triacsins	Other	25%
Region 71.1	siderophore	1	8702	desferrioxamine E	Other	75%
Region 91.1	lanthipeptide-class-iv	3240	26,017	labyrinthopeptin A2/labyrinthopeptin A1/labyrinthopeptin A3	RiPP:Lanthipeptide	40%
Region 93.1	NRPS	1	28,814	catenulisporolides	NRP + Polyketide	5%
Region 135.1	lanthipeptide-class-iii	1	11,872	catenulipeptin	RiPP:Lanthipeptide	60%

Region, contig of the draft genome; NRPS, Non-ribosomal peptide synthetase cluster; RiPP, ribosomally synthesized and post-translationally modified peptide product (RiPP) cluster; RRE-containing, RRE-element containing cluster; saccharide, Saccharide cluster (loose strictness, likely from primary metabolism); T1PKS, Type I PKS (Polyketide synthase); T2PKS, Type II PKS; T3PKS, Type III PKS.

**Table A6:** Distribution and loci of β-oxidation related putative genes in the draft genome of *Microtetraspora* sp. AC03309 based on RAST server analysis.

Transcriptional Regulator, TetR Family on the Same Contig as *lcp* Gene				
	Contig	Start (bp)	Stop (bp)	Length (bp)
1	contig000003	19,522	18,260	1263
2	contig000003	28,599	29,279	681

**Twin-Arginine Translocation Protein, TAT Protein (TatA, TatB, TatC)**				
	**Contig**	**Start (bp)**	**Stop (bp)**	**Length (bp)**
1	contig000002	137,788	137,363	426
2	contig000113	18,270	18,629	360
3	contig000133	9169	9507	339
4	contig000133	9535	10,389	855

**Mammalian Cell Entry, Mce (Mce1D, Mce1D)**				
	**Contig**	**Start (bp)**	**Stop (bp)**	**Length (bp)**
1	contig000186	1797	1339	459
2	contig000629	96	284	189
**Acyl-CoA synthase**				
	**Contig**	**Start (bp)**	**Stop (bp)**	**Length (bp)**
1	contig000004	157,269	155,719	1551
2	contig000004	152,549	150,963	1587
3	contig000033	8187	6544	1644

**Acyl-CoA Dehydrogenase (EC 1.3.8.1) (EC 1.3.8.7) (Putative)**				
	**Contig**	**Start (bp)**	**Stop (bp)**	**Length (bp)**
1	contig000016	19,917	20,870	954
2	contig000016	24,758	23,676	1083
3	contig000038	54,006	55,709	1704
4	contig000038	55,738	57,438	1701
5	contig000063	22,059	23,198	1140
6	contig000070	24,723	23,602	1122

**2,4-dienoyl-CoA Reductase, NADH: Flavin Oxidoreductases, Old Yellow Enzyme Family**				
	**Contig**	**Start (bp)**	**Stop (bp)**	**Length (bp)**
1	contig000070	29,836	28,598	1239

**Enoyl-CoA Hydratases (EC 4.2.1.17)**				
	**Contig**	**Start (bp)**	**Stop (bp)**	**Length (bp)**
1	contig000004	157,510	158,325	816
2	contig000008	7568	6855	711
3	contig000008	11,332	12,129	798
4	contig000013	81,498	80,584	915
5	contig000014	109,166	109,984	819
6	contig000016	7177	8541	1365
7	contig000016	14,015	13,206	810
8	contig000029	64,705	66,807	2103
9	contig000030	66,724	65,951	774
10	contig000034	65,180	64,380	801
11	contig000049	15,615	14,776	840
12	contig000066	20,230	20,994	765
13	contig000084	24,228	25,004	777
14	contig000113	12,043	11,240	804

**3-hydroxyacyl-CoA Dehydrogenases (EC 1.1.1.35)**				
	**Contig**	**Start (bp)**	**Stop (bp)**	**Length (bp)**
1	contig000001	58,250	56,769	1482
2	contig000008	27,541	26,771	771
3	contig000008	35,830	36,684	855
4	contig000016	5912	5157	756
5	contig000029	64,705	66,807	2103
6	contig000628	289	86	204

**3-ketoacyl-CoA Thiolase (EC 2.3.1.16)**				
	**Contig**	**Start (bp)**	**Stop (bp)**	**Length (bp)**
1	contig000001	49,954	48,761	1194
2	contig000002	54,042	52,825	1218
3	contig000005	108,324	109,448	1125
4	contig000008	19,025	17,337	1689
5	contig000008	34,593	35,762	1170
6	contig000008	70,364	69,177	1188
7	contig000009	12,987	11,806	1182
8	contig000025	61,696	62,844	1149
9	contig000029	63,524	64,708	1185
10	contig000033	3101	1878	1224
11	contig000033	6551	5379	1173
12	contig000034	68,356	67,148	1209
13	contig000049	17,108	18,265	1158
14	contig000066	45,751	46,896	1146
15	contig000090	24,745	23,573	1173

**α-Methylacyl-CoA Racemase (Mcr) (EC 5.1.99.4)**				
	**Contig**	**Start (bp)**	**Stop (bp)**	**Length (bp)**
1	contig000016	8557	9753	1194
2	contig000016	110,010	111,122	1113
3	contig000032	61,506	60,388	1119

**Superoxide Dismutase**				
	**Contig**	**Start (bp)**	**Stop (bp)**	**Length (bp)**
1	contig000020	73,292	72,687	606
2	contig000056	23,468	23,869	402

## Appendix B

**Table A7:** *Dactylosporangium* sp. AC04546 CRISPR array detected using CRISPRFinder server.

	Contig	Start (bp)	Stop (bp)
1	Contig000003	116,444	121,041
2	Contig000003	136,865	140,549
3	Contig000003	140,628	140,897
4	Contig000003	140,978	141,433
5	Contig000003	158,732	160,550
6	Contig000003	167,167	169,203
7	Contig 000002	176,434	176,695
8	Contig000031	68,133	68,314
9	Contig000051	75,685	76,012
10	Contig000103	11,150	11,388
11	Contig000106	41,651	41,808

**Table A8:** *Dactylosporangium* sp. AC04546 antibiotic resistant genes identified from CARD database.

	Contig	Start (bp)	Stop (bp)	Length (bp)	Classification	Identity (%)
1	Contig000013	85,766	85,392	375	aminoglycoside (ARO:3003395)	83
2	Contig000013	82,643	81,450	1194	glycopeptide antibiotic (ARO:3005036)	89

**Table A9:** *Dactylosporangium* sp. AC04546 drug target gene identified using PATRIC server, verified using DrugBank server.

	Contig	Product	Start (bp)	Stop (bp)	Length (bp)	Query Coverage	Identity (%)
1	contig00024	Cell division protein FtsZ, ftsZ	41,193	42,296	386	85	83
2	contig00121	RecA protein, recA	1566	517	1050	93	88

**Table A10:** *Dactylosporangium* sp. AC04546 urease and urea transporter putative genes identified using RAST server.

	Contig	Start (bp)	Stop (bp)	Length (bp)	Function
1	contig000144	11,078	11,380	303	Urease gamma subunit (EC 3.5.1.5) UreA
2	contig000144	11,377	11,682	306	Urease beta subunit (EC 3.5.1.5) UreB
3	contig000144	11,679	13,388	1710	Urease alpha subunit (EC 3.5.1.5) UreC
4	contig000144	13,391	14,050	660	Urease accessory protein UreF
5	contig000144	13,391	14,050	660	Urease accessory protein UreF
6	contig000144	13,391	14,050	660	Urease accessory protein UreF
7	contig000144	14,040	14,738	699	Urease accessory protein UreG
8	contig000144	14,040	14,738	699	Urease accessory protein UreG
9	contig000144	14,735	15,394	660	Urease accessory protein UreD
10	contig000144	14,735	15,394	660	Urease accessory protein UreD
11	contig000003	213,232	214,527	1296	Urea ABC transporter, substrate-binding protein UrtA
12	contig000003	214,640	215,524	885	Urea ABC transporter, permease protein UrtB
13	contig000003	215,521	216,615	1095	Urea ABC transporter, permease protein UrtC
14	contig000003	216,612	217,448	837	Urea ABC transporter, ATPase protein UrtD
15	contig000003	217,450	218,133	684	Urea ABC transporter, ATPase protein UrtE
16	contig000174	8890	9240	351	Urea ABC transporter, ATPase protein UrtE

**Table A11:** *Dactylosporangium* sp. AC04546 secondary metabolite biosynthetic gene clusters (BGCs) were predicted using AntiSMASH 6.0.

Region	Type	From	To	Most Similar Known Cluster		Similarity
Region 7.1	other	1	32,122			
Region 7.2	butyrolactone	181,275	189,513	colabomycin E	Polyketide:Type II	4%
Region 9.1	betalactone	127,964	156,777			
Region 23.1	LAP	63,471	86,862			
Region 28.1	terpene	63,486	89,541	hopene	Terpene	46%
Region 29.1	NRPS	1	53,614	gobichelin A/gobichelin B	NRP	27%
Region 33.1	redox-cofactor	47,552	69,629	lankacidin C	NRP + Polyketide	20%
Region 38.1	lanthipeptide-class-iv	1	17,570	salinilactam	Polyketide	8%
Region 47.1	RiPP-like	1	5832			
Region 52.1	thioamide-NRP	23,205	83,117	netropsin	NRP	27%
Region 58.1	redox-cofactor	54,961	76,748			
Region 62.1	NAPAA	27,298	61,263			
Region 79.1	terpene	39,001	58,687	isorenieratene	Terpene	25%
Region 81.1	lassopeptide	19,124	41,635	siamycin I	RiPP	28%
Region 87.1	T2PKS	1	51,425	arenimycin A	Polyketide:Type II + Saccharide:Hybrid/tailoring	68%
Region 90.1	terpene	35,800	49,030	calicheamicin	Polyketide	5%
Region 99.1	T3PKS	20,966	45,958	alkyl-O-dihydrogeranyl-methoxyhydroquinones	Terpene + Polyketide	57%
Region 118.1	NRPS	8823	36,925	A54145	NRP	3%
Region 229.1	siderophore	253	6171			

Region, contig of the draft genome; NRPS, Non-ribosomal peptide synthetase cluster; RiPP, ribosomally synthesized and post-translationally modified peptide product (RiPP) cluster; RRE-containing, RRE-element containing cluster; saccharide, Saccharide cluster (loose strictness, likely from primary metabolism); T1PKS, Type I PKS (Polyketide synthase); T2PKS, Type II PKS; T3PKS, Type III PKS.

**Table A12:** Distribution and loci of β-oxidation related putative genes in the draft genome of *Dactylosporangium* sp. AC04546.

Twin-Arginine Translocation Protein, TAT Protein (TatA, TatB, TatC)				
	Contig	Start (bp)	Stop (bp)	Length (bp)
1	contig000009	102,753	102,487	267
2	contig000071	21,417	20,446	972
3	contig000071	21,698	21,432	267
4	contig000128	29,800	29,369	432

**Acyl-CoA Synthase (EC 6.2.1.1)**				
	**Contig**	**Start (bp)**	**Stop (bp)**	**Length (bp)**
1	contig000098	31,823	33,694	1872
2	contig000151	21,809	19,845	1965

**Transcriptional Regulator, TetR Family on the Same Contig as lcp Gene**				
	**Contig**	**Start (bp)**	**Stop (bp)**	**Length (bp)**
1	contig000001	362,871	363,434	564
2	contig000082	7433	8032	600
3	contig000082	41,434	40,763	672
4	contig000082	43,443	42,790	654

**Acyl-CoA Dehydrogenase (EC 1.3.8.1) (EC 1.3.8.8) (Putative)**			
**Contig**	**Start (bp)**	**Stop (bp)**	**Length (bp)**
contig000001	308,373	309,527	1155
contig000002	210,799	211,995	1197
contig000002	226,160	224,997	1164
contig000006	79,214	80,359	1146
contig000006	91,911	93,056	1146
contig000006	109,177	110,382	1206
contig000006	123,342	124,502	1161
contig000007	32,267	31,008	1260
contig000007	42,815	43,978	1164
contig000008	111,357	112,454	1098
contig000009	125,869	124,712	1158
contig000010	136,785	135,604	1182
contig000010	151,583	150,426	1158
contig000015	106,328	105,189	1140
contig000015	125,651	124,686	966
contig000015	126,787	125,651	1137
contig000022	41,492	42,652	1161
contig000026	55,545	54,331	1215
contig000031	60,451	59240	1212
contig000032	93,065	91,926	1140
contig000039	4910	3720	1191
contig000039	26,773	27,924	1152
contig000043	24,574	26,259	1686
contig000058	40,472	41,617	1146
contig000058	44,004	45,245	1242
contig000070	60,103	61,281	1179
contig000079	56,242	57,411	1170
contig000079	57,408	58,457	1050
contig000082	5300	6439	1140
contig000082	39,644	40,777	1134
contig000095	11,194	12,378	1185
contig000095	12,375	13,523	1149
contig000102	18,437	17,304	1134
contig000108	19,517	20,767	1251
contig000116	8097	6244	1854
contig000121	6633	5491	1143
contig000128	19,042	17,828	1215
contig000154	11,134	9848	1287
contig000154	12,217	11,150	1068
contig000154	13,405	12,230	1176
contig000157	3155	4306	1152
contig000202	7053	8201	1149
contig000218	2054	126	1929

**2,4-dienoyl-CoA Reductase (EC 1.3.1.34)**				
	**Contig**	**Start (bp)**	**Stop (bp)**	**Length (bp)**
1	contig000007	126,818	125,733	1086

**Enoyl-CoA Hydratases (EC 4.2.1.17)**				
	**Contig**	**Start (bp)**	**Stop (bp)**	**Length (bp)**
1	contig000001	296,029	295,283	747
2	contig000001	300,636	301,421	786
3	contig000001	307,165	306,362	804
4	contig000001	329,756	330,586	831
5	contig000001	344,214	344,978	765
6	contig000002	202,945	202,163	783
7	contig000002	206,845	205,931	915
8	contig000006	81,885	81,088	798
9	contig000006	89,484	90,293	810
10	contig000006	97,828	96,953	876
11	contig000006	114,743	11,3946	798
12	contig000006	118,886	118,125	762
13	contig000006	120,324	122,495	2172
14	contig000006	124,537	125,340	804
15	contig000008	131,053	131,946	894
16	contig000009	130,569	131,345	777
17	contig000015	104,062	103,208	855
18	contig000017	101,441	100,656	786
19	contig000019	132,075	132,860	786
20	contig000025	2819	2001	819
21	contig000025	43,741	42,917	825
22	contig000027	47,243	48,025	783
23	contig000028	22,979	23,818	840
24	contig000028	26,480	28,549	2070
25	contig000031	14,833	12,764	2070
26	contig000032	69,957	70,736	780
27	contig000032	90,750	89,941	810
28	contig000039	8258	8986	729
29	contig000039	12,002	11,265	738
30	contig000039	33,697	34,668	972
31	contig000039	35,986	36,708	723
32	contig000043	48,021	50,117	2097
33	contig000045	37,575	36,832	744
34	contig000047	5992	5237	756
35	contig000048	27,562	26,795	768
36	contig000070	58,232	59,101	870
37	contig000070	61,278	62,096	819
38	contig000082	28,779	30,890	2112
39	contig000095	10,175	9405	771
40	contig000095	20,285	21,049	765
41	contig000095	30,027	29,176	852
42	contig000114	7605	8369	765
43	contig000174	16,732	17,184	453
44	contig000227	3486	2695	792

**3-Hydroxyacyl-CoA Dehydrogenases (EC 1.1.1.35)**				
	**Contig**	**Start (bp)**	**Stop (bp)**	**Length (bp)**
1	contig000017	39,722	41,509	1788
2	contig000018	104,278	105,594	1317
3	contig000095	18,898	18,137	762
4	contig000095	21,871	21,101	771

**Thiolase/3-Ketoacyl-CoA Thiolase (EC 2.3.1.16)**				
	**Contig**	**Start (bp)**	**Stop (bp)**	**Length (bp)**
1	contig000001	189,682	190,872	1191
2	contig000001	318,428	317,205	1224
3	contig000001	337,505	336,360	1146
4	contig000006	78,027	79,214	1188
5	contig000006	119,071	120,294	1224
6	contig000015	116,318	115,128	1191
7	contig000015	118,590	119,738	1149
8	contig000017	68,346	69,533	1188
9	contig000018	104,185	102,911	1275
10	contig000027	53,803	54,987	1185
11	contig000028	25,272	26,474	1203
12	contig000031	16,023	14,830	1194
13	contig000032	86,429	85,236	1194
14	contig000032	97,872	95,914	1959
15	contig000043	46,807	48,018	1212
16	contig000058	45,434	46,648	1215
17	contig000082	27,565	28,776	1212
18	contig000095	20,128	18,941	1188
19	contig000105	40,143	38,899	1245
20	contig000007	41,311	42,423	1113
21	contig000039	18,566	19,750	1185

**α-Methylacyl-CoA Racemase (Mcr) (EC 5.1.99.4)**				
	**Contig**	**Start (bp)**	**Stop (bp)**	**Length (bp)**
1	contig000001	326,963	328,114	1152
2	contig000027	43,150	41,963	1188
3	contig000032	91,899	90,757	1143
4	contig000082	3034	1871	1164
5	contig000095	15,365	16,525	1161
