# Supplementary material for: Rubber Degrading Strains: Microtetraspora and Dactylosporangium
Source: Polymers (Basel). 2021 Oct 13;13(20):3524. doi: 10.3390/polym13203524 (PMC8538451; doi:10.3390/polym13203524)
Supplement: Supplementary file 1 [file polymers-13-03524-s001.zip › Supplementary Figure 1.pdf]

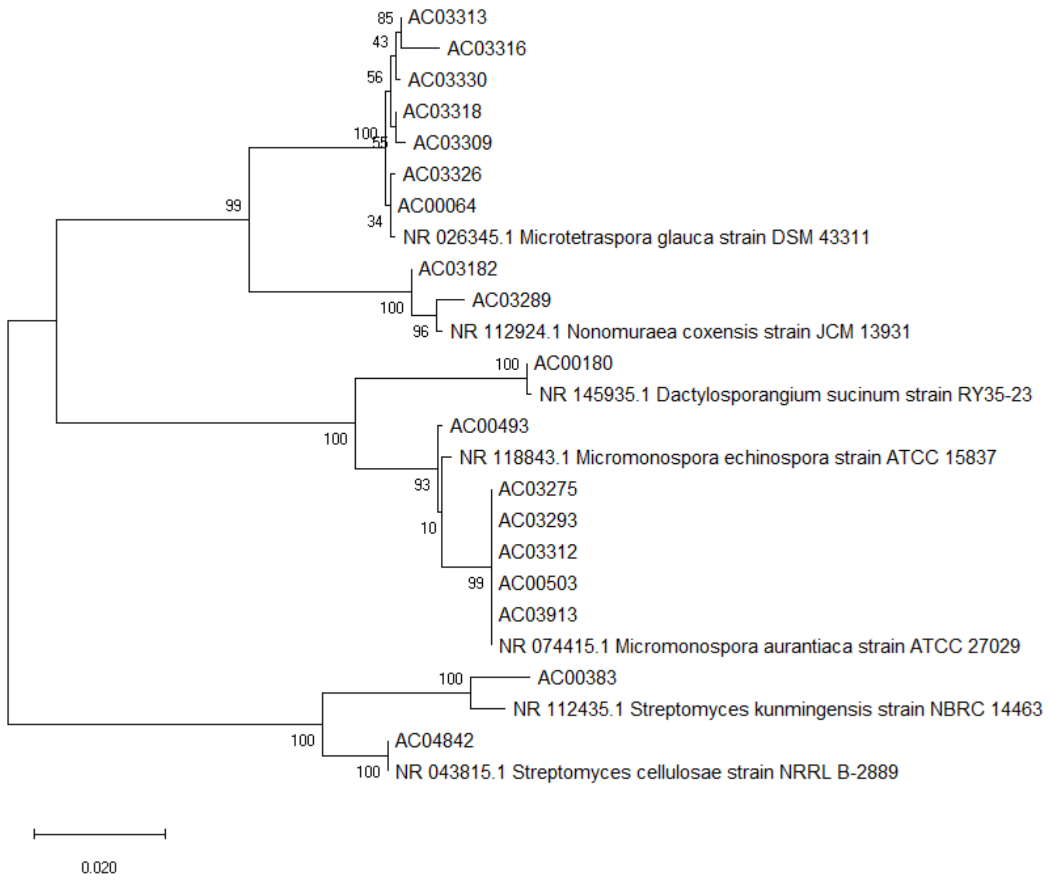

Figure S1. Clear zone forming strains 16S rRNA evolutionary relationships of taxa together with top 1 blast homology in GenBank database. The tree was constructed using MEGA X. The Bootstrap value was 1000. The bar below the phylogenetic trees represents the scale of sequence divergence.
