# Supplementary material for: Rubber Degrading Strains: Microtetraspora and Dactylosporangium
Source: Polymers (Basel). 2021 Oct 13;13(20):3524. doi: 10.3390/polym13203524 (PMC8538451; doi:10.3390/polym13203524)

Supplementary Figure S2. Latex clearing protein (lcp) amino acid sequence alignment of the 18 clear zone forming strains to determine the 13-residue long highly conserved region.

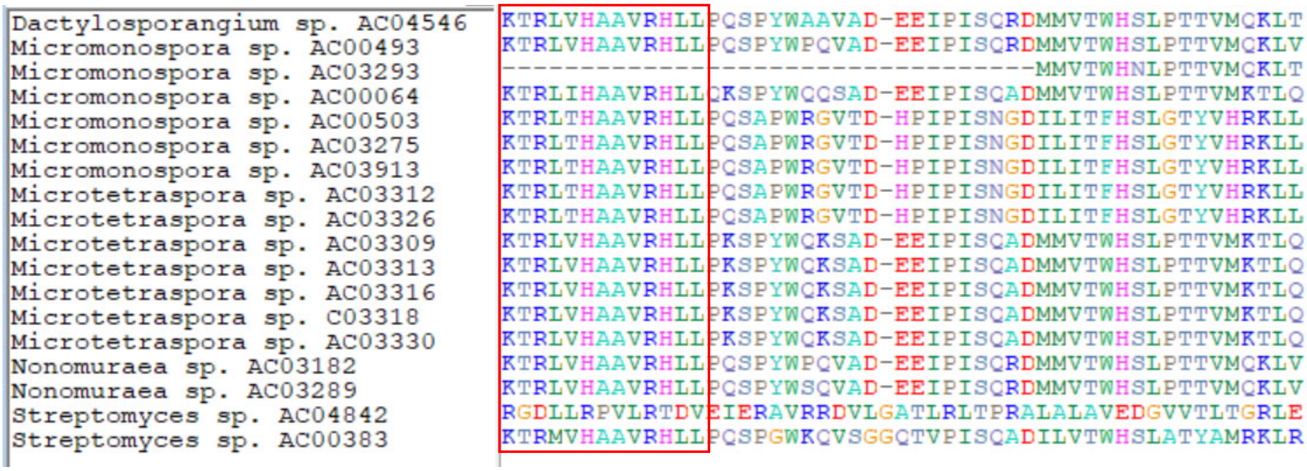

Supplement: Supplementary file 1 [file polymers-13-03524-s001.zip › Supplementary Figure 2.pdf]
